# RNA-seq analysis-based study on the effects of gestational diabetes mellitus on macrosomia

**DOI:** 10.3389/fendo.2024.1330704

**Published:** 2024-04-10

**Authors:** Qianqian Gao, Guanying Xu, Guijie Wang, Wei Wang, Chao Zhu, Yang Shi, Changzhuang Guo, Jing Cong, Hongxia Ming, Dongmei Su, Xu Ma

**Affiliations:** ^1^ Shandong Engineering Research Center of Novel Pharmaceutical Excipients, Sustained and Controlled Released Preparations, Dezhou, Shandong, China; ^2^ Omics Technologies and Health Engineering Research Center, Dezhou, Shandong, China; ^3^ College of Medicine and Nursing, Dezhou University, Dezhou, China; ^4^ Department of Obsterics and Gynecology, Dezhou Maternal and Child Health Hospital, Dezhou, China; ^5^ Department of Ecology and Environmental Protection, Linyi Vocational College of Science and Technology, Linyi, China; ^6^ College of Life Science, Dezhou University, Dezhou, China; ^7^ College of Ecology, Resources and Environment, Dezhou, China; ^8^ Graduate School of Peking Union Medical College, Chinese Academy of Medical Sciences, Beijing, China; ^9^ Department of Genetics, Key Laboratory of Reproductive Health Engineering Technology Research of China’s National Health Commission, Beijing, China

**Keywords:** macrosomia, hyperglycemia, placenta, differentially expressed genes, hub genes

## Abstract

**Background:**

Both the mother and the infant are negatively impacted by macrosomia. Macrosomia is three times as common in hyperglycemic mothers as in normal mothers. This study sought to determine why hyperglycemic mothers experienced higher macrosomia. Methods: Hematoxylin and Eosin staining was used to detect the placental structure of normal mother(NN), mothers who gave birth to macrosomia(NM), and mothers who gave birth to macrosomia and had hyperglycemia (DM). The gene expressions of different groups were detected by RNA-seq. The differentially expressed genes (DEGs) were screened with DESeq2 R software and verified by qRT-PCR. The STRING database was used to build protein-protein interaction networks of DEGs. The Cytoscape was used to screen the Hub genes of the different group.

**Results:**

The NN group’s placental weight differed significantly from that of the other groups. The structure of NN group’s placenta is different from that of the other group, too. 614 and 3207 DEGs of NM and DM, respectively, were examined in comparison to the NN group. Additionally, 394 DEGs of DM were examined in comparison to NM. qRT-PCR verified the results of RNA-seq. Nucleolar stress appears to be an important factor in macrosomia, according on the results of KEGG and GO analyses. The results revealed 74 overlapped DEGs that acted as links between hyperglycemia and macrosomia, and 10 of these, known as Hub genes, were key players in this process. Additionally, this analysis believes that due of their close connections, non-overlapping Hubs shouldn’t be discounted.

**Conclusion:**

In diabetic mother, ten Hub genes (RPL36, RPS29, RPL8 and so on) are key factors in the increased macrosomia in hyperglycemia. Hyperglycemia and macrosomia are linked by 74 overlapping DEGs. Additionally, this approach contends that non-overlapping Hubs shouldn’t be ignored because of their tight relationships.

## 1 Introduction

Macrosomia is typically defined as a birth weight above the 90th percentile for gestational age or >4,000 g. Gestational diabetes mellitus is a state of hyperglycemia that occurs during pregnancy. Macrosomia has a number of negative impacts on both moms and infants (1). Hyperglycemia can result in serious maternal and newborn problems, which are a growing source of health anxiety (2). When compared to controls with normal glucose levels, about 15–45% of infants born to diabetic moms may develop macrosomia, which is a 3-fold greater rate. More studies are proving that aberrant placenta development and function are related to pregnancy problems and poor fetal outcomes associated with hyperglycemia (3). In order to reduce macrosomia, it is necessary to understand the process through which hyperglycemia causes macrosomia.

Previous research has attempted to identify the reason why gestational diabetes mellitus (GDM) suffers from higher macrosomia. According to metabolic profile of carnitine metabolism in second trimester GDM women, Carnitine metabolism aberration could predict macrosomia complicated with GDM (4). Reduced maternal adiponectin and higher IGF-1 levels in the placenta of GDM women may have increased GLUT-1 expression through enhanced insulin/IGF-1 signaling, which may have affected fetal growth (5). With a normal pre-pregnancy BMI, the development of GDM-induced macrosomia is tightly correlated with fasting plasma glucose and placenta. The mechanism may be hyperglycemia promotes trophoblast cell proliferation via ERK1/2 signaling (6).

By examining the gene expression of macrosomia and hyperglycemia combined with macrosomia, we intended to investigate the reason why there is greater macrosomia in hyperglycemia in the current study. Three groups of clinic samples were created. We collected the placentas from NN, NM, and DM. H&E staining was used to reveal their structural details. The placentas from the three groups were subjected to RNA-seq. In order to identify the DEGs between NN and NM, NN and DM, and NM and DM, the following thresholds were used: p<0.05 and |log2 (fold change)|>1. qRT-PCR was used to validate several DEGs. We examined the functional and route enrichment of DEGs to better investigate the connection between hyperglycemia and macrosomia. The CytoHubba in Cytoscape plug-in was used to aid in the selection of the Hub genes. To investigate the mechanism of macrosomia brought on by hyperglycemia, the overlapping DEGs and the protein-protein interaction (PPI) between Hub genes from different comparisons were investigated.

## 2 Materials and methods

### 2.1 Patients

The department of obstetrics and gynecology of Maternal and Child Health Centre in Dezhou recruited the subjects. After receiving informed consent, placental tissue samples were collected for this investigation, which was authorized by the institutional review board. Ages of the expectant mothers ranged from 25 to 40. The chosen control women had no relevant medical history and no difficulties from pregnancy. Newborns were weighed right after delivery. According to Endocrine Society standards, hyperglycemia was diagnosed when fasting blood glucose was > 5.1 mmol/L. When the birth weight exceeded 4,000 grams, macrosomia was identified.

### 2.2 Tissue collection

Placentas were obtained from 30 healthy women, 15 women with macrosomia and 15 women with hyperglycemia and macrosomia immediately after caesarean section, some immediately frozen in liquid nitrogen for RNA extraction, and some immobilized in formaldehyde for H&E staining.

### 2.3 Placentas morphology

Placentas tissue (n=10) from each group were fixed in 4% formaldehyde, embedded in paraffin, and sectioned at 4 µm thickness for visualization. H&E staining was used to observe the sections under a microscope (Nikon, Eclipse).

### 2.4 RNA library construction and high-throughput sequencing

Following the manufacturer’s instructions, total RNA was extracted from placentas using Trizol Reagent (Invitrogen: 15596018). Utilizing the Agilent RNA Nano 6000 Assay Kit and the 2100 Bioanalyzer instrument, RNA purity and quantity were assessed (Agilent Technologies, Santa Clara, CA, USA). Following the manufacturer’s instructions, total RNA was used as input material for the RNA sample preparations. Briefly, mRNA was purified from total RNA by using poly-T oligo-attached magnetic beads. Fragmentation was carried out using divalent cations under elevated temperature in First Strand Synthesis Reaction Buffer(5X). First strand cDNA was synthesized using random hexamer primer and M-MuLV Reverse Transcriptase, then use RNaseH to degrade the RNA. Second strand cDNA synthesis was subsequently performed using DNA PolymeraseI and dNTP. Remaining overhangs were converted into blunt ends via exonuclease/polymerase activities. After adenylation of 3’ ends of DNA fragments, Adaptor with hairpin loop structure were ligated to prepare for hybridization. In order to select cDNA fragments of preferentially 370~420bp in length, the library fragments were purified with AMPure XP system (Beckman Coulter, Beverly, USA). Then PCR amplification, the PCR product was purified by AMPure XP beads, and the library was finally obtained. In order to ensure the quality of the library, the library needs to be tested. After the construction of the library, the library was initially quantified by Qubit2.0 Fluorometer, then diluted to 1.5ng/ul, and the insert size of the library is detected by Agilent 2100 bioanalyzer. After insert size meets the expectation, qRT-PCR is used to accurately quantify the effective concentration of the library (the effective concentration of the library is higher than that of 2nM) to ensure the quality of the library.

After the library is qualified, the different libraries are pooling according to the effective concentration and the target amount of data off the machine, then being sequenced by the Illumina NovaSeq 6000. The end reading of 150bp pairing is generated. The basic principle of sequencing is to synthesize and sequence at the same time (Sequencing by Synthesis). Four fluorescent labeled dNTP, DNA polymerase and splice primers were added to the sequenced flow cell and amplified. When the sequence cluster extends the complementary chain, each dNTP labeled by fluorescence can release the corresponding fluorescence. The sequencer captures the fluorescence signal and converts the optical signal into the sequencing peak by computer software, so as to obtain the sequence information of the fragment to be tested.

### 2.5 Transcriptome data analysis

The image data measured by the high-throughput sequencer are converted into sequence data (reads) by CASAVA base recognition. Raw data (raw reads) of fastq format were firstly processed through in-house perl scripts. In this step, clean data (clean reads) were obtained by removing reads containing adapter, reads containing Nbase and low quality reads from raw data. At the same time, Q20, Q30 and GC content the clean data were calculated. All the downstream analyses were based on the clean data with high quality.

Reference genome and gene model annotation files were downloaded from genome website directly. Index of the reference genome was built using Hisat2(v2.0.5) and paired-end clean reads were aligned to the reference genome usingHisat2 (v2.0.5). We selected Hisat2 as the mapping tool for that Hisat2 can generate a database of splice junctions based on the gene model annotation file and thus a better mapping result than other non-splice mapping tools.

The mapped reads of each sample were assembled by StringTie (v1.3.3b) (Mihaela Pertea.et al. 2015) in a reference-based approach. StringTie uses a novel network flow algorithm as well as an optional *de novo* assembly step to assemble and quantitate full length transcripts representing multiple splice variants for each gene locus.

The feature Counts v1.5.0-p3 was used to count the reads numbers mapped to each gene. And then FPKM of each gene was calculated based on the length of the gene and reads count mapped to this gene. FPKM, expected number of Fragments Per Kilobase of transcript sequence per Millions base pairs sequenced, considers the effect of sequencing depth and gene length for the reads count at the same time, and is currently the most commonly used method for estimating gene expression levels.

Differential expression analysis of two groups (more than three biological replicates per group) was performed using the DESeq2 R package (1.20.0). DESeq2 provide statistical routines for determining differential expression in digital gene expression data using a model based on the negative binomial distribution. The resulting P-values were adjusted using the Benjamini and Hochberg’s approach for controlling the false discovery rate. padj&lt;=0.05 and |log2(foldchange)| &gt;= 1 were set as the threshold for significantly differential expression. For the data downloaded from GEO database (GSE203346 and GSE154414), differential expression analysis of two groups (more than three biological replicates per group) was performed using the limma R package and selected with the same standard.

### 2.6 RNA extraction and qRT-PCR analysis

According to the manufacturer’s instructions, total RNA was isolated from the placentas using Trizol reagent (Life Technologies). The Evo M-MLV reverse transcription kit (Accurate Biotechnology (Hunan) Co., Ltd.) was used for reverse transcription (RT-PCR). SYBR Green Pro Taq HS premixed qPCR kit from Accurate Biotechnology (Hunan) Co., Ltd. was used for the quantitative PCR. Reactions were conducted with 1μL RT-PCR cDNA, 0.5 μL forward and reverse primers (10 μmol/L), 8μL water and 10 μL SYBR Green. Each reaction was normalized by co-amplification of β-actin. The samples were run by the StepOne real-time PCR machine (ABI, USA). The primers used in this study were *ATP5ME* forward 5′- CGCGCTACAATTACCTAAAA -3′ and reverse 5′- ATATGCTGTCATCTTCTGCC -3′; *COX5B* forward 5´- TTGGGAAAAGCTGTCTGTTA -3´ and reverse 5´- GTCCCATTCATTGCATTACG -3´; *RPL35* forward 5′- AAGCTCTCTAAGATCCGAGTC -3′and reverse 5′- GCTTGTACTTCTTGCCCTTG -3′; *RPL37A* forward 5´- AAACGTACCAAGAAAGTCGG -3´ and reverse5´- CAGCTCGTCTCTTCATCTTG -3´ and β-actin forward 5´-GTCCACCTTCCAGCAGATGT-3’ and reverse 5´-TCACCTTCACCGTTCCAGTT-3´.

### 2.7 GO and KEGG analysis of DEGs

Gene Ontology (GO) enrichment analysis of differentially expressed genes was implemented by the clusterProfiler R package (3.8.1), in which gene length bias was corrected. GO terms with corrected Pvalue less than 0.05 were considered significantly enriched by differential expressed genes. KEGG is a database resource for understanding high-level functions and utilities of the biological system, such as the cell, the organism and the ecosystem, from molecular-level information, especially large-scale molecular datasets generated by genome sequencing and other high-through put experimental technologies (http://www.genome.jp/kegg/). We used clusterProfiler R package (3.8.1) to test the statistical enrichment of differential expression genes in KEGG pathways.

### 2.8 Seek hub genes and the PPI enrichment analysis of them

The STRING database is a search engine for interacting genes that seeks to build PPI networks of various genes based on known and projected PPIs and examine the proteins that interact with one another (7). PPIs of DEGs of NM and DM were generated using the web tool STRING, and the confidence score (>0.4) was used as the screening criteria. Cytoscape software was then used to visualize the PPI network (version 3.7.2).CytoHubba was used to find Hub genes. Degree of CytoHubba plug-in was used to select the top 40 genes with the highest node connection closeness as the Hub genes (8).

The PPI enrichment analysis of Hub genes from two groups was constructed at STRING database (https://string-db.org/). MCODE plug-in (Node Score Cutoff: 0.2 Haircut: true Fluff: false K-Core: 2 Max. Depth from Seed: 100) was used to calculate accurate correlation level as well as identifying essential PPI network modules (9). Additionally, other Cytoscape add-ins namely, CytoHubba and CytoNCA were used to identify the network’s highest linkage Hub genes (10).

### 2.9 Statistical analysis

The Kruskal Wallis test and *T*-test were used to calculate the statistical significance of the experimental data. Bonferroni-corrected P values to correct for account comparisons. The significance level was set as ** p < 0.01. Error bars denote standard deviations. The correlation between fetal weight and placenta weight was explored using the Spearman’s rank correlation coefficient test.

## 3 Results

### 3.1 Hyperglycemia affected the weight of the placenta

The results of Kruskal Wallis test between three groups in seven variants, there are four significantly differences, which are pregestational BMI, Glucose, fetal Birth weight and placenta weight. The result of Kruskal Wallis test between two groups showed that significant differences in BMI occurred between the NN group and the DM group(P <0.001), significant differences in glucose content in blood occurred between the DM group and the other two group(both P <0.01), significant differences in fetal birth weight occurred between the NN group and the other two groups(both P <0.001), significant differences in placenta weight occurred between the NN group and the other two groups (both P <0.001). Perhaps because the sample size was not large enough, there is no significant difference in BMI between NN group and NM group Compared with the NM group, the DM placenta weight rose by 6.3%, but the change is not significant (p>0.05).The weight of the fetus has a positive relationship with placental weight (**Table 1**). The results of the double-digit correlation analysis of placenta and fetal weight are displayed in **Table 2**. The Spearman’s rho is 0.762, p<0.01.

**Table 1:** Clinical and analytical characteristics of the cohort.

	NN(n=30)	NM(n=15)	DM(n=15)	P value+
Maternal age (years)	32.29 ± 1.55	31.35 ± 3.29	34.14 ± 4.99	0.126
Pregestational BMI(kg/m^2^)	38.81 ± 6.49	42.19 ± 7.25	44.3 ± 4.78**	0.001
Gestational weight gain (kg)	16.71 ± 6.97	16.13 ± 7.79	12.56 ± 4.24	0.106
Glucose(mg/dL)	4.54 ± 0.29	4.55 ± 0.82	6.17 ± 1.32**	0.000
Gestational delivery (weeks)	38.65 ± 0.19	38.74 ± 0.94	38.39 ± 1.09	0.503
Fetal birth weight (g)	3337.79 ± 313.75	4150 ± 159.86**	4221.42 ± 253.97**	0.000
Placental weight (g)	580.8 ± 93.89	734.31 ± 130.072**	780.02 ± 164.05**	0.000

**P-value<0.01vs control.

**Table 2:** Correlation analysis.

	Fetal_weight	Placenta_weight
Fetal_weight	1	
Placenta_weight	.762^**^	1

**Correlation is significant at the 0.01 level (2-tailed).

### 3.2 Structure of placentas

**Figure 1:**
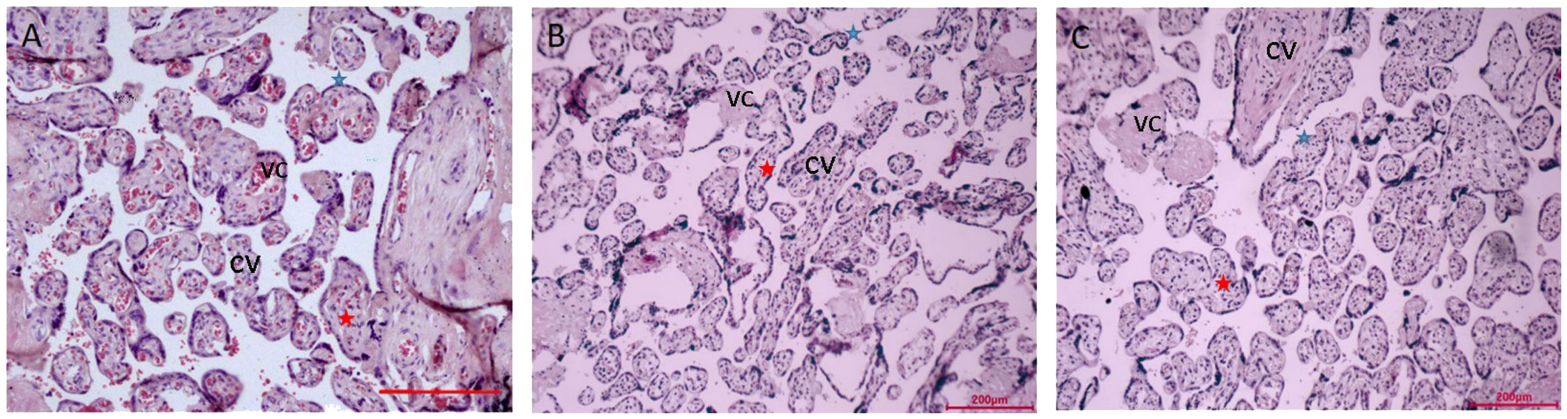
Microscopy of the placentas. **(A)** Low power view with the chorionic plate of normal pregnancy (38+ 6 weeks gestation). **(B)** Low power view with the chorionic plate of normal pregnancy with macrosomia (39+ 1 weeks gestation). **(C)** Low power view with the chorionic plate of hyperglycemia with macrosomia (38+ 3weeks gestation). Bar=200µm. CV, chorionic villi; VC, vascular congestion; blue star is syncytiotrophoblasts, red star is cytotrophoblastic cell.

The villi’s size is uniform throughout the placenta tissue of the NN group (**Figure 1A**). Villi don’t have any breaks or damage. The well-developed syncytial trophoblasts that make up the surface layer of the placental villi are dispersed in a flat, single-layer configuration, and the free surface has morphological rules, uniform distribution, and neatly aligned microvilli that are finger-shaped.

In the surface layer of the placenta villi of the NM placenta, the syncytial trophoblasts were arranged in a monolayer row (**Figure 1B**). In the placenta of extravillous and swelling villi have more concentrated deposits of fibrinoid. Syncytial trophoblasts are dispersed throughout the DM placenta’s placental villi (**Figure 1C**). Villi come in various sizes. Red blood cells can be visible in the vascular lumen, the capillary lumen is intact, and some endothelial cells swell. The lumen of capillary endothelial cells has significantly shrunk and is clearly constricted.

### 3.3 Transcriptome assembly and annotation

RNA-Seq was used to compare the transcriptomic landscapes of NM verse NN, DM verse NN and DM verse NM placentas. All the samples sequenced on the Illumina HiSeq X platform produced about 43.4, 44.6 and 46.6 million raw reads for NN, NM and DM samples, respectively, covering 6.37, 6.21 and 6.69 GB of sequence data, respectively. The NN group received 42.45 million clean reads as a result of over 97% of the raw reads surviving quality and trimming. With almost 92% of the raw reads surviving quality checks and trimming, the NM and DM groups, respectively, produced 41.4 and 44.6 million clean reads. **Supplementary Table 1** lists many characteristics, including average read size, Q30 percentage, and others. The genome was mapped using clean reads for the ensuing analysis.

### 3.4 Identification of differently expressed genes

**Figure 2:**
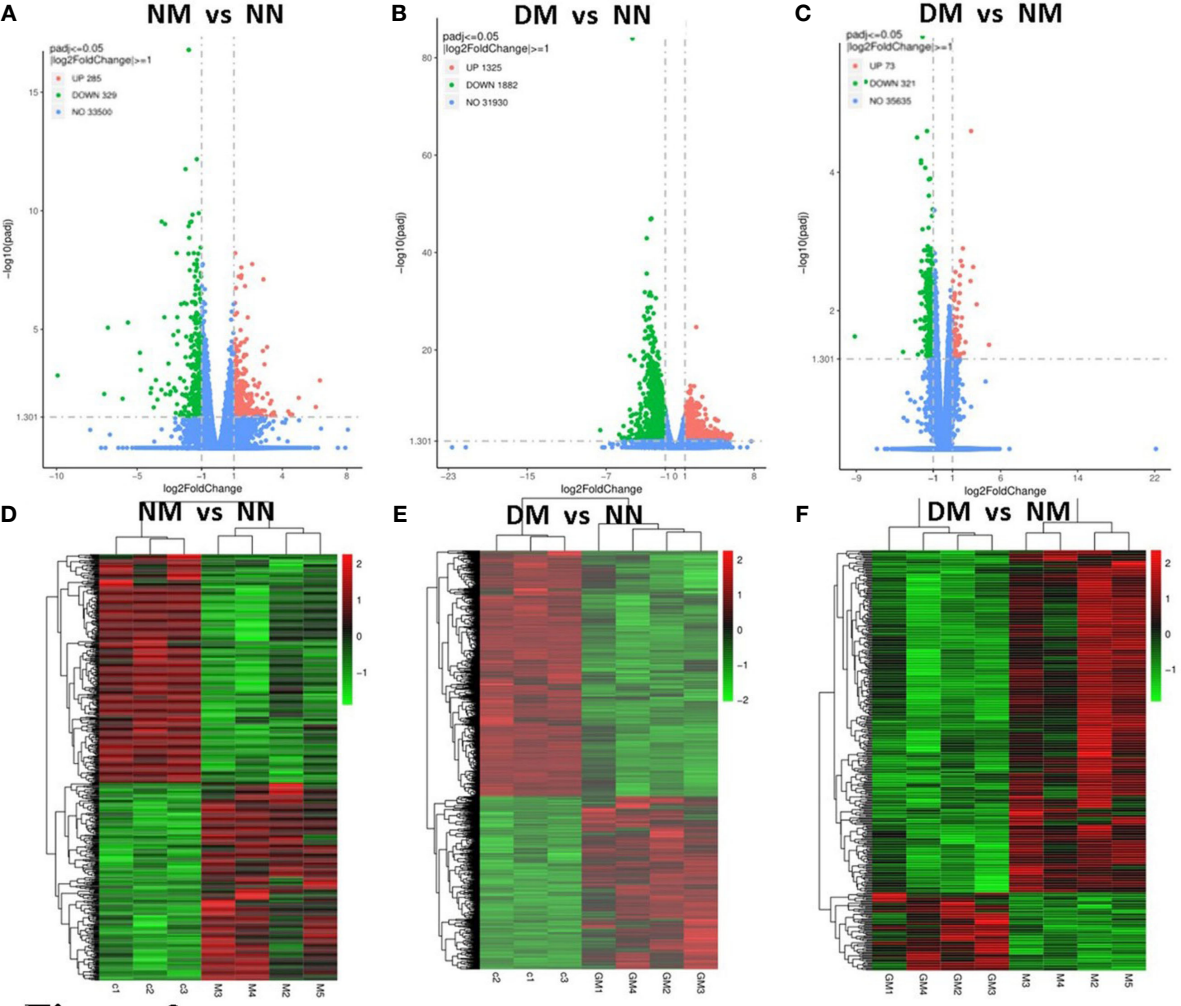
Identification of differential expressed genes in the placenta from macrosomia group and hyperglycemia with macrosomia. Volcano plots were used to display differential expressed RNAs of NM versus NN **(A)**, DM versus NN **(B)** and DM versus NM in term placenta**(C)**. **(D)** Heatmaps were used to display expressed RNAs of NM versus NN **(D)**, DM versus NN **(E)** and DM versus NM in term placenta **(F)**. NN, control group; NM, macrosomia; DM, hyperglycemia with macrosomia.

We conducted a clustering analysis between the NN and NM, DM group based on the levels of gene expression. For comparative and enrichment analysis of DEGs, we defined genes with ∣log2fold∣ changes>1 and padj<0.05 as significantly differently expressed genes. The volcano plot analysis also showed significant DEGs NM versus NN (**Figure 2A**), DM versus NN (**Figure 2B**) and DM versus NM (**Figure 2C**). The up-regulated DEGs are represented by red dots, while the down-regulated DEGs are represented by green dots. In the NM versus NN group, a total of 614 genes showed differential expression, with 285 up-regulated and 329 down-regulated DEGs (**Supplementary Table 2**). In the DM versus NN group, 3207 genes were differentially expressed, with 1325 up-regulated and 1882 down-regulated DEGs (**Supplementary Table 2**). In the DM versus NM group, 394 genes were differentially expressed, with 73 up-regulated and 321 down-regulated DEGs. Additionally, the heatmap showed the placenta genes that were up-regulated in red and down-regulated in green in NM versus NN (**Figure 2D**), DM versus NN (**Figure 2E**), and DM versus NM (**Figure 2F**).

### 3.5 mRNA expression patterns were verified via qRT-PCR

**Figure 3:**
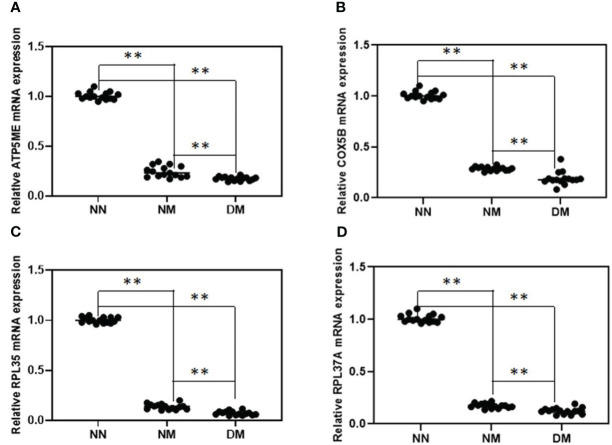
Relative expression analysis of four selected DEGs between NN and NM and DM group. qRT-PCR was used to analyze of the mRNA expression of ATP5ME **(A)**, COX5B **(B)**, RPL35 **(C)** and RPL37A **(D)** in term placenta between NN and NM and DM (n=15) respectively β-actin was used as the internal reference. **P-value<0.01.

Following confirmation using the gene-specific primers described in the procedure and qRT-PCR of the NM and DM group (n = 15) vs control group (n = 15), we discovered that the change direction of these four genes is consistent with the RNA-seq data. The results showed that the four randomly selected genes reduced in abnormal groups significantly. In comparison to the NN group, the expression of the genes for ATP5ME, COX5B, RPL35, and RPL37A was considerably lower in NM and DM (p< 0.01)(**Figure 3**). Their expression in the DM group was also lower than in the NM group, which were statistically significant (p< 0.01)

### 3.6 GO and KEGG analysis of DEGs

**Figure 4:**
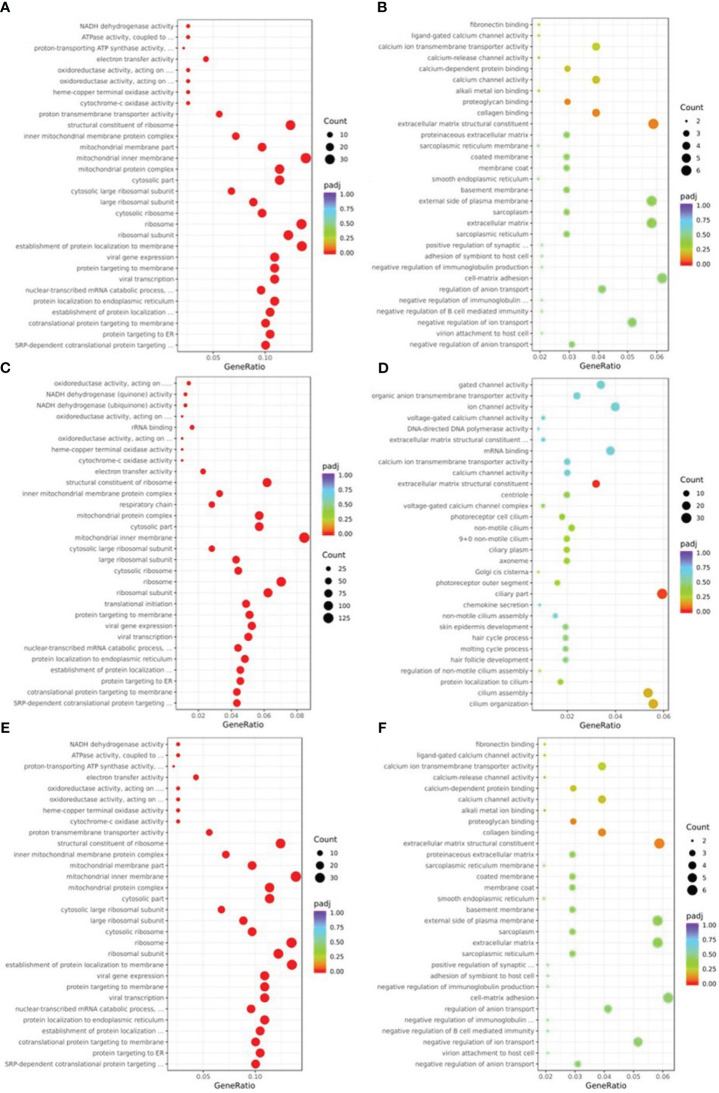
Gene ontology (GO) classification of differentially expressed genes (DEGs). **(A)** GO annotation showed that down-regulated DEGs of NM versus NN were associated with different biological processes, cell component and molecular functions. **(B)** GO annotation showed that up-regulated DEGs of NM versus NN were associated with different biological processes, cell component and molecular functions. **(C)** GO annotation showed that down-regulated DEGs of DM versus NN were associated with different biological processes, cell component and molecular functions. **(D)** GO annotation showed that up-regulated DEGs of DM versus NN were associated with different biological processes, cell component and molecular functions. **(E)** GO annotation showed that down-regulated DEGs of DM versus NM were associated with different biological processes, cell component and molecular functions. **(F)** GO annotation showed that up-regulated DEGs of DM versus NM were associated with different biological processes, cell component and molecular functions.

GO analysis using terminology related to biological process, cellular component, and molecular function was used to define the function of DEGs. As seen in **Figure 4**, the biological processes that were primarily impacted by the down-regulated DEGs of NM (**Figure 4A**) were protein targeting, nucleoside monophosphate metabolism, and oxidative phosphorylation. The ribosome, the mitochondrial inner membrane, and the respiratory chain were the three major areas where the DEGs of NM involved in the cellular component were down-regulated. The NM DEGs that were down-regulated primarily included ribosome structural components, proton transmembrane transporter activity, and oxidoreductase activity in molecular functions. The GO analysis of up-regulated DEGs in NM had poor enrichment (p>0.05) (**Figure 4B**). The biological processes that were primarily impacted by the down-regulated DEGs of DM versus NN (**Figure 4C**) involved protein targeting, RNA catabolism, and ribonucleotide metabolism. The DM DEGs that were down-regulated primarily implicated the cytosol, inner membrane of the mitochondria, and ribosomes in the cellular component. The down-regulated DEGs of DM vs NN involved in molecular functions were primarily linked to cadherin binding, electron transfer activity, and ribosome structural components. The biological processes that were engaged in the up-regulated DEGs of DM vs NN (**Figure 4D**) were primarily connected to cilium organization, blood circulation, and epithelial cell proliferation. The ciliary portion and the extracellular matrix structural constituent was the primary area where the up-regulated DEGs of DM versus NN engaged in the cellular component were most closely associated. The mitochondrial inner membrane, ribosome, and mitochondrial matrix were the primary components of the down-regulated DEGs of DM versus NM (**Figure 4E**) engaged in the cellular component. The molecular functions that the down-regulated DEGs of DM versus NM were involved in were primarily connected to ribosome structural components, electron transfer activity, and protein kinase inhibitor activity. The biological process-related up-regulated DEGs of DM versus NM were primarily connected to the response to xenobiotic stimuli. The GO analysis of the up-regulated DEGs of DM versus NM (**Figure 4F**) had poor enrichment.

**Figure 5:**
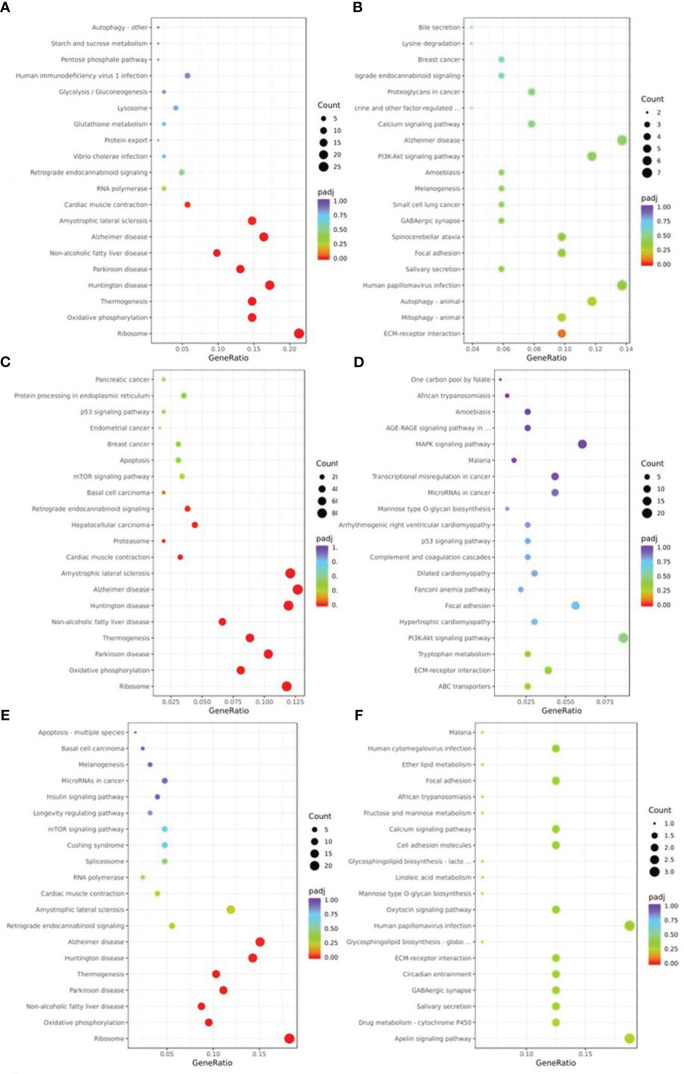
KEGG pathway classification of differentially expressed genes (DEGs) KEGG pathway analysis showed that DEGs were involved in different signaling pathways. **(A)** KEGG pathway analysis of the down-regulated DEGs of NM versus NN. **(B)** KEGG pathway analysis of the up-regulated DEGs of NM versus NN were associated with different biological processes, cell component and molecular functions. **(C)** KEGG pathway analysis of the down-regulated DEGs of DM versus NN were associated with different biological processes, cell component and molecular functions. **(D)** KEGG pathway analysis of the up-regulated DEGs of DM versus NN were associated with different biological processes, cell component and molecular functions. **(E)** KEGG pathway analysis of the down-regulated DEGs of DM versus NM were associated with different biological processes, cell component and molecular functions. **(F)** KEGG pathway analysis of the up-regulated DEGs of DM versus NM were associated with different biological processes, cell component and molecular functions.

The pathways of DEGs were predicted using KEGG pathway analysis. As shown in **Figure 5**, the down-regulated DEGs in NM versus NN (**Figure 5A**) were primarily focused on ribosome (about 26 DEGs, including RPL37A and RPL35), Alzheimer’s disease (about 20 DEGs, including SNCA and COX8A), and retrograde endocannabinoid signaling (about 6 DEGs, such as NDUFA1 and GNG5). The ECM-receptor interaction (about 5 DEGs, including FN1 and TNC) the primary areas of up-regulated DEGs in NM versus NN (**Figure 5B**). The down-regulated DEGs in DM versus NN (**Figure 5C**) were primarily focused on ribosome (approximately 80 DEGs, including RPL37 and RPS11), Alzheimer’s disease (about 86 DEGs), and cardiac muscle contraction (about 22 DEGs, such as UQCRQ and TNNC1). The KEGG analysis of up-regulated DEGs in DM versus NN (**Figure 5D**) had poor enrichment. As shown in **Figure 5E**, the DEGs that were down-regulated in DM compared to NM were primarily related to the ribosome (about 23 DEGs, including RPL37A and RPS11), Alzheimer’s disease (about 19 DEGs, including APC2 and COX8A), and non-alcoholic fatty liver disease (about 11 DEGs, such as SNCA and COX8A). In the KEGG analysis (**Figure 5F**), the up-regulated DEGs in DM versus NM had poor enrichment.

### 3.7 PPI hub genes identification

**Figure 6:**
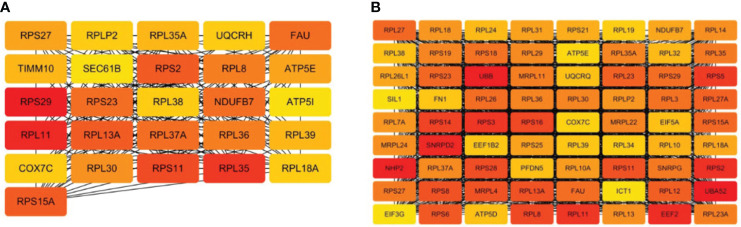
Hub genes of macrosomia and hyperglycemia with macrosomia. **(A)** the Hub genes of macrosomia, **(B)** the Hub genes of hyperglycemia with macrosomia. The color of the key gene represents its degree. The higher the degree of gene, the more redd her color.

To mine the Hub genes of NM and DM, the DEGs were loaded into string and MC>0.4 was cutoffs. A network consisted of 354 nodes and 1164 edges with p< 1.0e-16 were obtained in NM versus NN. The Hub gene was chosen from the PPI network using the CytoHubba plug-in and Degree method, as illustrated in **Figure 6A** and **Table 3**. All 26 genes were downregulated. The score for the last Hub gene is 58. Because String can only analyze the limit of 2000 proteins, we further analyzed the named DEGs in DM versus NN with ∣log2fold∣ changes>1.12 with String, which got a network with 1676 nodes, 9131 edges and PPI enrichment p-value <1.0e-16. The Hub gene was illustrated in **Figure 6B**. All of the Hub genes of DM group are down-regulated. The score of the last Hub gene is 162. In the DM group, these 72 genes showed reduced expression. The Hub genes of the NM group were included in the Hub gene of the DM group except TIMM10, UQCRH, SEC61B and ATP5I.

**Table 3:** Changes of hub genes in two groups.

Group	Gene ID	Degree	Change
NM	RPS29	82	down
NM	RPL11	80	down
NM	RPL35	78	down
NM	RPS11	76	down
NM	RPS2	74	down
NM	RPS15A	74	down
NM	RPL13A	74	down
NM	NDUFB7	72	down
NM	RPL37A	72	down
NM	RPS23	72	down
NM	FAU	72	down
NM	RPL36	70	down
NM	RPL8	70	down
NM	RPL30	66	down
NM	ATP5ME	64	down
NM	RPS27	64	down
NM	RPL39	62	down
NM	RPL35A	62	down
NM	TIMM10	62	down
NM	UQCRH	60	down
NM	COX7C	60	down
NM	RPLP2	60	down
NM	RPL18A	60	down
NM	RPL38	60	down
NM	SEC61B	58	down
NM	ATP5I	58	down
DM	UBA52	326	down
DM	UBB	280	down
DM	NHP2	254	down
DM	SNRPD2	248	down
DM	EEF2	244	down
DM	RPS2	238	down
DM	RPS3	236	down
DM	RPL11	236	down
DM	RPS5	230	down
DM	RPS14	226	down
DM	RPL8	224	down
DM	RPS16	224	down
DM	RPS28	222	down
DM	MRPL4	220	down
DM	RPS11	220	down
DM	RPL13A	220	down
DM	RPS8	220	down
DM	RPS6	216	down
DM	RPL12	214	down
DM	RPL23	210	down
DM	RPL27A	210	down
DM	RPS23	208	down
DM	RPS15A	208	down
DM	RPL27	208	down
DM	RPS29	206	down
DM	FAU	206	down
DM	RPL26	206	down
DM	RPL3	204	down
DM	RPS18	204	down
DM	RPL35	202	down
DM	RPS27	202	down
DM	SNRPG	202	down
DM	RPL23A	200	down
DM	MRPL11	200	down
DM	RPL36	198	down
DM	RPS19	198	down
DM	RPL30	198	down
DM	MRPL22	198	down
DM	RPL35A	198	down
DM	RPL29	196	down
DM	RPL37A	196	down
DM	RPL13	192	down
DM	RPL14	192	down
DM	MRPL24	192	down
DM	RPL7A	190	down
DM	RPLP2	190	down
DM	RPL18	190	down
DM	RPL10A	188	down
DM	RPS25	186	down
DM	RPL31	186	down
DM	RPL18A	184	down
DM	NDUFB7	184	down
DM	RPL26L1	184	down
DM	RPS21	182	down
DM	RPL10	180	down
DM	RPL32	178	down
DM	RPL24	178	down
DM	ATP5D	176	down
DM	EIF5A	176	down
DM	UQCRQ	176	down
DM	PFDN5	176	down
DM	RPL38	176	down
DM	FN1	174	down
DM	RPL39	174	down
DM	EEF1B2	174	down
DM	RPL19	170	down
DM	RPL34	170	down
DM	COX7C	166	down
DM	ATP5E	164	down
DM	SIL1	162	down
DM	EIF3G	162	down
DM	ICT1	162	down

### 3.8 Exploration the relationship between macrosomia and hyperglycemia by DEGs

**Figure 7:**
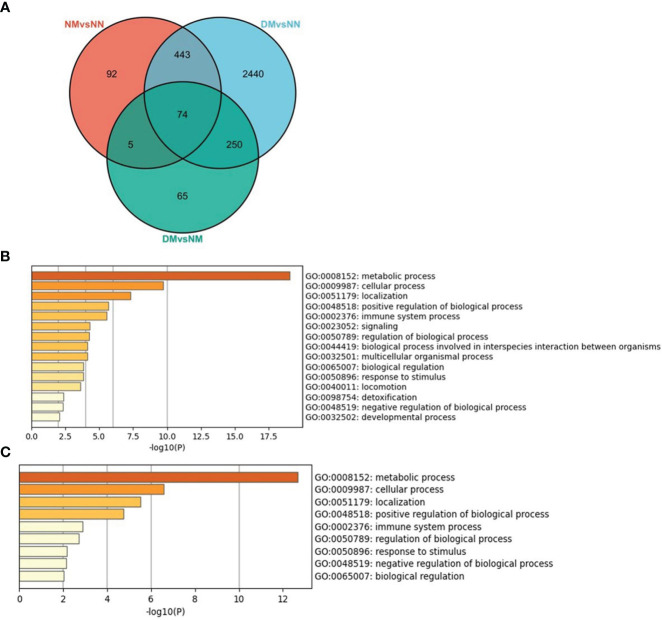
The overlapped DEGs of three sets of crossover genes with different alignments. **(A)** the overlapped DEGs of three sets of crossover genes with different alignments, **(B)** GO annotation of the overlapped DEGs of NM versus NN and DM versus NN. **(C)** GO annotation of the overlapped DEGs of three sets of crossover genes with different alignments. The color of the key gene represents its degree. The higher the degree, the more redder her color.

We scanned the DEGs of NM versus NN, DM versus NN, and DM versus NM and studied the overlapped DEGs between two and three comparisons (**Figure 7A**). There were 517 DEGs overlapped in NM and DM groups, which changed in the same direction, containing 299 down-regulated DEGs and 218 up-regulated DEGs. That is to say, more than 84% of DEGs in the NM group changed in the same direction in the DM group. These genes involved in the biological process were mainly related to metabolic process, cellular process, signaling and so on (**Figure 7B**). There are 79 genes overlapped in NM versus NN and DM versus NM including 10 Hub genes of NM group. The 74 overlapped DEGs of three groups involved in the biological process were mainly related to metabolic process, cellular process and positive regulation of biological process (**Figure 7C**). These 74 DEGs includes RPS29, RPL35, RPS11, RPS2, NDUFB7, RPL37A, FAU, RPL36, RPL8 and RPL18A, which are Hub genes in macrosomia. From this, we infer that hyperglycemia changes the expressions of these Hub genes and promotes the incidence of macrosomia.

### 3.9 Exploration the relationship between hyperglycemia and macrosomia by hub genes

**Figure 8:**
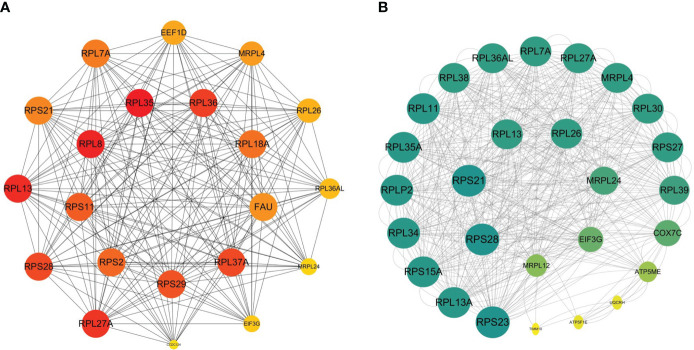
Relations between hyperglycemia with macrosomia and macrosomia. **(A)** The Hub genes of DM versus NM, **(B)** the PPI between the Hub genes of DM vs NM and NM vs NN except the overlapped Hub genes. The color of the key gene represents its degree. The higher the degree of the gene, the greener her color.

We acquired a network consisted of 303 nodes and 724 edges with p< 1.0e-16 in DM versus NM. The cytoHubba plug-in and the Degree algorithm were used to select the Hub gene from the PPI network as shown in **Figure 8A**. All of these 21 genes were down-regulated. Furthermore, RPS29, RPL35, RPS11, RPS2, NDUFB7, RPL37A, FAU, RPL36, RPL8 and RPL18A are Hub genes of NM versus NN. Except MRPL12 and CCDC124, the other Hub genes were included in the Hub gene of the DM group. We then analyzed the relationship between the other Hub gene in the DM versus NM group and the Hub gene in the NM versus NN group (**Figure 8B**). This result shows that in addition to the overlapped Hub gene, there is a strong link between the Hub genes in the DM versus NM group.

## 4 Discussion

Hyperglycemia represents the most common form of altered glucose in pregnant women, and may cause macrosomia, hypertension and cardiovascular disease (11). Pregnant women with hyperglycemia through the placenta will stimulate the production of a large amount of insulin secretion of the fetus to be able to make full use of blood sugar, and promote the synthesis of protein and fat, so that the fetus grows larger (12). This study aims to provide light on the gene expression level of the diabetic macrosomia mechanism. 614 DEGs were found using RNA-seq in the placenta of pregnant women who delivered macrosomia. 3207 DEGs were found in the placenta of pregnant women with macrosomia who also had high blood sugar levels throughout pregnancy. In comparison to NM, 394 DEGs were discovered in DM. Four of DEGs were verified by qRT-PCR.

In order to analyze the pathogenesis of macrosomia, hyperglycemic macrosomia and the impact of hyperglycemia on macrosomia, KEGG and GO were used to analyze the differential genes in the three groups of alignment. Through GO analysis, it was discovered that NM DEGs are involved in protein targeting, nucleoside monophosphate metabolism, oxidative phosphorylation, cell-matrix adhesion, cellular calcium ion homeostasis, and nutritional response. The results of the GO analysis showed that DM DEGs are involved in the targeting of proteins, RNA catabolism, ribonucleotide metabolism, cilium organization, blood circulation, and proliferation of epithelial cells. According to GO analysis, DM versus NM DEGs were found to be involved in protein targeting, the electron transport chain, oxidative phosphorylation, and the response to xenobiotic stimulation. The KEGG results and GO results were not entirely in agreement. In three comparisons, the down-regulated DEGs in the ribosome overlapped. The substantial downregulation of ribosomal genes in this study implies that ribosome anomalies play a significant role in the development of macrosomia. A diverse array of disorders’ etiology is due to defects in ribosome biosynthesis and function (13). Nucleolar stress results from the ribosome genesis process being disturbed. Fat formation is a result of nucleolar stress (14).

In this work, the intersection of DEGs in three sets of different alignments was further studied. According to the results, there is a significant relationship between DM and NM since more than 84% of the DEGs in the NM group altered in the same direction as those in the DM group. In comparison to NM versus NN alignment, 74 DEGs in the DM versus NM alignment changed in the same way. This means they are linking genes between hyperglycemia and macrosomia. Three sets of distinct alignments contain 74 DEGs that have had their orientation reversed, indicating that they are significant in hyperglycemia and macrosomia.

Cytoscape was used to evaluate the Hub genes of the DM and NM groups in order to investigate the fundamental cause of increased macrosomia in hyperglycemia. Among the 26 Hub genes in the NM group, 22 genes were DM group Hub genes, and 10 genes were DM group versus NM group. These ten genes are located in the DEGs that overlap in three different sets of alignments. This leads us to hypothesize that these 10 genes are crucial linkers between hyperglycemia and macrosomia. They were all deregulated. Except NDUFB7, the other proteins are ribosome protein. Ribosome impairment is important in obesity (15).

RPS29 induced apoptosis, which means the downregulation of RPS29 is associated with cell proliferation (16). In GSE154414, it was likewise downregulated. RPL35 encodes a ribosomal protein that is a component of the 60S subunit. RPL35 was revealed as putative key drivers of stress granules (17). RPS11encodes a member of the S17P family of ribosomal proteins that is a component of the 40S subunit. RPS11 is also a stress response marker (18). RPS2 plays a critical role in the regulation of p53 signaling including the ribosomal stress response (19). RPL37A encodes a ribosomal protein that is a component of the 60S subunit. It related pathways including peptide chain elongation and rRNA processing in the nucleus and cytosol (20). FAU encodes a fusion protein consisting of the ubiquitin-like protein fubi at the N terminus and ribosomal protein S30 at the C terminus. Processing FAU is required for 40S maturation and depends on USP36 (21). RPL36 encodes a ribosomal protein that is a component of the 60S subunit (22). RPL8 overexpression enhances apoptosis brought on by FasL (23). RPL18A encodes a member of the L18AE family of ribosomal proteins that is a component of the 60S subunit (24). Confusion in the “production and processing” of ribosomal RNA can cause nucleolar stress (25).

This investigation also constructed PPI between DM versus NM and NM versus NN non-overlapping Hub genes and revealed that there is an unbreakable link between them, further illuminating the association between hyperglycemia and macrosomia. So, we shouldn’t ignore their connection while exposing additional macrosomia in hyperglycemia. Ribosomal proteins including RPL18 maintain the identity of mouse embryonic stem cells (mESCs) and regulate the expression of 2C transcripts through a unique RP-RPL11-MDM2-P53-DUX cascade (26). RPL11 encodes a ribosomal protein that is a component of the 60S subunit. RPL11 promotes the active of p53, which can induce apoptosis (27). If these genes were verified in maternal blood in the future, they maybe biomarker in clinical practice. We can also consider increasing the expression of these genes to reduce the occurrence of macrosomia

## 5 Conclusions

In conclusion, we first detected the placenta’s aberrant structure. After that, using RNA-seq analysis, the team investigated the molecular causes of aberrant placental morphological structures. The team then used GO and KEGG to analyze the internal mechanism of macrosomia and hyperglycemia. Then, the team analyzed the reasons for the high incidence of macrosomia in hyperglycemia from the perspectives of overlapping differential genes and Hub genes. The results showed 74 genes that served as bridges between hyperglycemia and macrosomia and the 10 Hub genes played a crucial part in this process. Also, it is the opinion of this work that non-overlapping Hubs should not be disregarded because of their close connections.

## Data availability statement

The datasets presented in this study can be found in online repositories. The names of the repository/repositories and accession number(s) can be found below: https://www.ncbi.nlm.nih.gov/, PRJNA853493.

## Ethics statement

The studies involving humans were approved by medical research ethics review committee of the Dezhou Maternal and Child Health Hospital. The studies were conducted in accordance with the local legislation and institutional requirements. The participants provided their written informed consent to participate in this study.

## Author contributions

QG: Conceptualization, Writing – original draft, Funding acquisition. GX: Writing – review & editing, Resources, Data curation. GW: Writing – review & editing, Resources, Investigation. WW: Writing – review & editing, Data curation. CZ: Writing – review & editing, Validation, Data curation. YS: Writing – review & editing, Investigation. CG: Writing – review & editing, Investigation. JC: Writing – review & editing, Resources, Investigation. HM: Writing – review & editing, Data curation. DS: Writing – review & editing, Funding acquisition. XM: Writing – review & editing, Supervision.
