# Supplementary material for: RNA-seq analysis-based study on the effects of gestational diabetes mellitus on macrosomia
Source: Front Endocrinol (Lausanne). 2024 Apr 10;15:1330704. doi: 10.3389/fendo.2024.1330704 (PMC11039845; doi:10.3389/fendo.2024.1330704)
Supplement: Supplementary file 1 [file Table_1.docx]

Supplementary table1

. Statistical results of raw and preprocessed sequences

| Sample | raw_reads | clean_reads | clean_bases | error_rate | Q20 | Q30 | GC_pct |
| --- | --- | --- | --- | --- | --- | --- | --- |
| NN1(c1) | 46723230 | 45506446 | 6.83G | 0.03 | 97.58 | 92.49 | 51.33 |
| NN2(c2) | 42104794 | 41247170 | 6.19G | 0.02 | 98.24 | 94.82 | 51.67 |
| NN3(c3) | 41388444 | 40602110 | 6.09G | 0.03 | 97.54 | 92.38 | 51.2 |
| NM1(M2) | 46341376 | 42877050 | 6.43G | 0.03 | 97.73 | 93.68 | 50.17 |
| NM2(M3) | 43717940 | 40078098 | 6.01G | 0.03 | 97.77 | 93.89 | 49.11 |
| NM3(M4) | 43169418 | 39370000 | 5.91G | 0.03 | 97.82 | 94.02 | 47.72 |
| NM4(M5) | 45247394 | 43387446 | 6.51G | 0.03 | 97.62 | 93.44 | 49.35 |
| DM1(GM1) | 41368844 | 39744824 | 5.96G | 0.03 | 97.73 | 93.57 | 47.03 |
| DM2(GM2) | 50275974 | 47987936 | 7.2G | 0.03 | 97.66 | 93.65 | 45.58 |
| DM3(GM3) | 47709342 | 45222142 | 6.78G | 0.03 | 97.71 | 93.54 | 44.79 |
| DM4(GM4) | 47046208 | 45329282 | 6.8G | 0.03 | 97.76 | 93.6 | 44.94 |
