# Supplementary material for: RNA-seq analysis-based study on the effects of gestational diabetes mellitus on macrosomia
Source: Front Endocrinol (Lausanne). 2024 Apr 10;15:1330704. doi: 10.3389/fendo.2024.1330704 (PMC11039845; doi:10.3389/fendo.2024.1330704)
Supplement: Supplementary file 2 [file Table_2.docx]

Supplementary Table 2 DEGs in NM, DM vs NN

| groups | directions | Gene ID |
| --- | --- | --- |
| NM vs NN | Down | ENSG00000239264, ENSG00000213741, ENSG00000112695, ENSG00000227081, ENSG00000139192, ENSG00000224877, ENSG00000163220, ENSG00000276168, ENSG00000156482, novel.1307, ENSG00000239857, ENSG00000186468, ENSG00000198356, ENSG00000244313, ENSG00000271303, ENSG00000218426, ENSG00000127184, ENSG00000175416, ENSG00000214253, ENSG00000122691, ENSG00000125652, ENSG00000006327, ENSG00000172428, ENSG00000130725, ENSG00000184840, ENSG00000197956, ENSG00000099203, ENSG00000134419, ENSG00000173457, ENSG00000175550, ENSG00000117410, ENSG00000177954, ENSG00000182768, ENSG00000168701, ENSG00000182512, ENSG00000100410, ENSG00000149806, ENSG00000109265, ENSG00000163993, ENSG00000137970, ENSG00000213866, ENSG00000181524, ENSG00000198258, ENSG00000197756, ENSG00000005075, ENSG00000129235, ENSG00000174307, ENSG00000214182, ENSG00000228253, ENSG00000241494, ENSG00000183617, ENSG00000124172, ENSG00000244187, ENSG00000002330, ENSG00000244716, ENSG00000253570, ENSG00000230291, ENSG00000173156, ENSG00000074842, ENSG00000162998, ENSG00000156603, ENSG00000204614, ENSG00000174156, ENSG00000100938, ENSG00000109475, ENSG00000104140, ENSG00000172336, ENSG00000166317, ENSG00000240342, ENSG00000106538, ENSG00000259032, ENSG00000136942, ENSG00000234797, ENSG00000106803, ENSG00000100216, ENSG00000243829, ENSG00000183876, ENSG00000166337, ENSG00000125356, ENSG00000123395, ENSG00000104979, ENSG00000103274, ENSG00000123144, ENSG00000127554, ENSG00000103363, ENSG00000160799, ENSG00000136867, ENSG00000139112, ENSG00000183011, ENSG00000180992, ENSG00000099795, ENSG00000166562, ENSG00000173207, ENSG00000198918, ENSG00000187808, ENSG00000101084, ENSG00000142541, ENSG00000169100, ENSG00000225178, ENSG00000283154, ENSG00000153093, ENSG00000278771, ENSG00000215845, ENSG00000167193, ENSG00000168040, ENSG00000174028, ENSG00000146677, ENSG00000135404, ENSG00000161677, ENSG00000173153, ENSG00000179085, ENSG00000197582, ENSG00000257084, ENSG00000182154, ENSG00000130545, ENSG00000182899, ENSG00000011600, ENSG00000220842, ENSG00000172809, ENSG00000184350, ENSG00000131495, ENSG00000145494, ENSG00000184508, ENSG00000236552, ENSG00000249456, ENSG00000228589, ENSG00000116586, ENSG00000272325, ENSG00000105373, ENSG00000178449, ENSG00000158716, ENSG00000092758, ENSG00000166595, ENSG00000106244, ENSG00000171223, ENSG00000198171, ENSG00000125995, ENSG00000133169, ENSG00000142534, ENSG00000115350, ENSG00000111775, ENSG00000179967, ENSG00000145592, novel.1476, ENSG00000185883, ENSG00000177600, ENSG00000170498, ENSG00000119013, ENSG00000165507, ENSG00000217716, ENSG00000135940, ENSG00000174917, ENSG00000233762, ENSG00000173113, ENSG00000134825, ENSG00000278845, ENSG00000128185, ENSG00000105640, ENSG00000237991, ENSG00000174021, ENSG00000103266, ENSG00000107223, ENSG00000161016, ENSG00000174109, ENSG00000175756, ENSG00000164182, ENSG00000122121, ENSG00000117862, ENSG00000212802, ENSG00000126432, ENSG00000170516, ENSG00000175315, ENSG00000173372, ENSG00000100605, ENSG00000229117, ENSG00000188612, ENSG00000140264, ENSG00000230202, ENSG00000176046, ENSG00000171402, ENSG00000226221, novel.649, ENSG00000278922, ENSG00000145335, ENSG00000112799, novel.689, ENSG00000164825, ENSG00000213218, ENSG00000139239, ENSG00000204922, ENSG00000228474, ENSG00000106367, ENSG00000167700, ENSG00000237506, ENSG00000196683, ENSG00000007520, ENSG00000099800, ENSG00000217130, novel.860, ENSG00000176101, ENSG00000124733, ENSG00000218819, ENSG00000233276, ENSG00000085265, ENSG00000095970, ENSG00000142676, ENSG00000106633, novel.1365, ENSG00000186007, ENSG00000132967, ENSG00000163344, novel.781, ENSG00000213862, ENSG00000165272, ENSG00000171224, ENSG00000240376, ENSG00000140988, ENSG00000007080, ENSG00000146540, novel.1200, ENSG00000156875, novel.984, ENSG00000173660, ENSG00000232320, ENSG00000134809, ENSG00000007255, ENSG00000171421, ENSG00000135363, ENSG00000136149, ENSG00000077942, ENSG00000110245, ENSG00000176340, ENSG00000092841, ENSG00000189129, ENSG00000205155, ENSG00000150779, ENSG00000122133, ENSG00000213442, ENSG00000160285, ENSG00000106123, ENSG00000100122, ENSG00000132507, ENSG00000197249, ENSG00000205426, ENSG00000267390, ENSG00000161653, ENSG00000197620, ENSG00000105404, ENSG00000136371, ENSG00000149925, ENSG00000163923, ENSG00000154319, ENSG00000151364, ENSG00000257315, novel.1167, ENSG00000100142, ENSG00000185834, ENSG00000152082, ENSG00000196976, ENSG00000105198, novel.770, ENSG00000168329, ENSG00000262814, ENSG00000241468, ENSG00000280071, ENSG00000139410, ENSG00000224183, ENSG00000169689, ENSG00000074211, ENSG00000111845, ENSG00000188856, novel.796, ENSG00000130748, novel.1219, ENSG00000147586, ENSG00000177096, ENSG00000213261, ENSG00000230330, ENSG00000155368, ENSG00000177700, ENSG00000001460, ENSG00000203724, ENSG00000286646, ENSG00000140743, ENSG00000176845, ENSG00000112667, ENSG00000111671, ENSG00000239405, ENSG00000226287, ENSG00000100628, novel.1587, ENSG00000160221, ENSG00000188910, ENSG00000127774, ENSG00000242071, ENSG00000169020, ENSG00000173272, ENSG00000225200, ENSG00000102309, ENSG00000203760, ENSG00000232389, novel.870, ENSG00000180871, ENSG00000185614, ENSG00000275074, ENSG00000139714, ENSG00000255262, ENSG00000107833, ENSG00000233937, ENSG00000230613, ENSG00000205078, ENSG00000213178, ENSG00000189280, ENSG00000187837, ENSG00000261512, ENSG00000092051, ENSG00000210140, novel.353, ENSG00000245112, ENSG00000261472, ENSG00000243273, ENSG00000197258, ENSG00000253958, ENSG00000130255, ENSG00000240087, ENSG00000259642, ENSG00000090382, ENSG00000198468, ENSG00000163815, |
|  | up | ENSG00000175455, novel.705, ENSG00000110455, ENSG00000069493, ENSG00000188676, ENSG00000215252, novel.1518, novel.1569, ENSG00000159708, ENSG00000114857, novel.1536, ENSG00000079134, ENSG00000230590, ENSG00000259972, ENSG00000206530, ENSG00000082175, ENSG00000253671, ENSG00000253939, ENSG00000224660, ENSG00000008311, ENSG00000240038, ENSG00000189423, ENSG00000143318, ENSG00000272645, ENSG00000132530, ENSG00000268205, ENSG00000244754, ENSG00000123201, ENSG00000231607, ENSG00000269235, ENSG00000264112, ENSG00000151012, ENSG00000113971, ENSG00000214548, ENSG00000232593, ENSG00000259820, ENSG00000246859, ENSG00000213066, ENSG00000269842, ENSG00000225213, ENSG00000223403, ENSG00000121454, ENSG00000267002, novel.919, ENSG00000271335, ENSG00000177990, ENSG00000176293, ENSG00000101104, ENSG00000267313, ENSG00000288569, ENSG00000176809, ENSG00000271533, ENSG00000075826, ENSG00000265298, ENSG00000229436, ENSG00000172061, ENSG00000196757, ENSG00000256667, ENSG00000242193, ENSG00000243716, ENSG00000082512, novel.1576, ENSG00000229036, ENSG00000113916, ENSG00000175967, ENSG00000242028, ENSG00000226476, ENSG00000283050, ENSG00000159086, ENSG00000100483, ENSG00000187653, ENSG00000285728, novel.562, ENSG00000225964, ENSG00000127241, ENSG00000071991, ENSG00000280077, ENSG00000108846, ENSG00000260296, ENSG00000124224, novel.255, ENSG00000177409, novel.149, ENSG00000249669, novel.809, ENSG00000131711, ENSG00000213139, ENSG00000249307, ENSG00000120458, ENSG00000178852, ENSG00000286207, novel.83, ENSG00000165810, ENSG00000248323, ENSG00000137801, ENSG00000204172, ENSG00000270055, ENSG00000227719, ENSG00000210194, ENSG00000285979, ENSG00000288065, ENSG00000261087, ENSG00000102287, ENSG00000198185, ENSG00000263272, ENSG00000230630, ENSG00000254206, ENSG00000106976, ENSG00000278768, ENSG00000260853, ENSG00000209082, ENSG00000104938, ENSG00000258168, ENSG00000230280, ENSG00000281026, ENSG00000243970, ENSG00000120322, ENSG00000106302, ENSG00000251562, ENSG00000242268, ENSG00000250596, ENSG00000226332, ENSG00000015479, ENSG00000115414, ENSG00000143127, ENSG00000081052, ENSG00000280099, ENSG00000213782, ENSG00000259668, ENSG00000270069, ENSG00000240591, ENSG00000279089, novel.941, ENSG00000233215, ENSG00000271895, ENSG00000264456, ENSG00000227954, ENSG00000169203, ENSG00000169926, ENSG00000223959, novel.420, ENSG00000198064, novel.1248, novel.1127, ENSG00000180139, ENSG00000271151, ENSG00000185495, ENSG00000279926, ENSG00000279059, novel.153, ENSG00000223519, ENSG00000109927, ENSG00000225670, ENSG00000152926, ENSG00000271870, ENSG00000263006, ENSG00000006016, ENSG00000258521, ENSG00000228486, novel.750, ENSG00000009724, ENSG00000167992, novel.904, ENSG00000261324, ENSG00000186567, ENSG00000235592, ENSG00000228393, ENSG00000228492, ENSG00000286545, ENSG00000253159, ENSG00000197683, ENSG00000129521, ENSG00000196912, ENSG00000244161, ENSG00000274383, ENSG00000286964, ENSG00000247982, ENSG00000258441, ENSG00000229967, ENSG00000153291, novel.587, novel.660, ENSG00000163611, ENSG00000167371, ENSG00000286699, ENSG00000159307, ENSG00000118473, ENSG00000239265, ENSG00000239704, ENSG00000238039, ENSG00000229152, ENSG00000280120, ENSG00000270589, ENSG00000135063, ENSG00000260361, ENSG00000171970, ENSG00000136002, ENSG00000248174, ENSG00000226312, ENSG00000238273, novel.448, ENSG00000281404, novel.526, ENSG00000232111, novel.899, ENSG00000180178, ENSG00000272871, ENSG00000245711, ENSG00000227790, ENSG00000270804, ENSG00000167766, ENSG00000279518, novel.249, ENSG00000166863, ENSG00000225313, ENSG00000272335, ENSG00000271553, novel.322, ENSG00000284747, ENSG00000046653, ENSG00000188869, ENSG00000157404, ENSG00000272356, ENSG00000229618, ENSG00000143624, ENSG00000055732, ENSG00000269053, ENSG00000213170, ENSG00000041982, ENSG00000118432, ENSG00000143452, ENSG00000288538, ENSG00000261556, ENSG00000138615, ENSG00000160781, ENSG00000271092, ENSG00000196208, ENSG00000183171, novel.1185, ENSG00000249715, ENSG00000198838, novel.533, ENSG00000174697, ENSG00000137959, ENSG00000179698, ENSG00000236714, novel.360, ENSG00000226985, ENSG00000116183, ENSG00000280247, ENSG00000111837, ENSG00000092529, ENSG00000225507, ENSG00000188338, ENSG00000237491, ENSG00000273142, ENSG00000268621, ENSG00000278959, ENSG00000165379, novel.1519, ENSG00000226091, ENSG00000259194, ENSG00000260528, ENSG00000166473, ENSG00000089737, ENSG00000254694, ENSG00000188933, novel.276, ENSG00000154645, ENSG00000251273, ENSG00000258057, ENSG00000227885, ENSG00000273373, novel.829, ENSG00000134245, ENSG00000226252, ENSG00000143869, novel.585, ENSG00000227934, ENSG00000238018, ENSG00000230869, ENSG00000122674, ENSG00000157765, ENSG00000259240, ENSG00000251495, |
| DM vs NN | Down | ENSG00000204525, ENSG00000272702, ENSG00000262165, ENSG00000204257, ENSG00000213435, ENSG00000170967, novel.1240, ENSG00000134873, ENSG00000280233, novel.838, ENSG00000266402, ENSG00000270006, novel.233, ENSG00000110203, ENSG00000204805, novel.1179, ENSG00000284395, ENSG00000283071, ENSG00000279140, ENSG00000286517, ENSG00000219451, ENSG00000180209, ENSG00000228797, ENSG00000104918, novel.1307, ENSG00000271869, ENSG00000230291, ENSG00000199753, novel.689, ENSG00000230613, ENSG00000284128, ENSG00000264443, ENSG00000219023, ENSG00000210140, ENSG00000186818, ENSG00000244086, ENSG00000276168, ENSG00000167476, ENSG00000262814, ENSG00000160318, ENSG00000170516, ENSG00000268678, ENSG00000165948, novel.1200, ENSG00000229413, ENSG00000143001, novel.129, ENSG00000173825, ENSG00000229368, ENSG00000210176, ENSG00000169704, ENSG00000160886, ENSG00000218819, ENSG00000164825, ENSG00000176125, ENSG00000198483, ENSG00000278771, ENSG00000272325, ENSG00000168282, ENSG00000225200, ENSG00000198797, ENSG00000287463, ENSG00000243517, ENSG00000257084, novel.1596, ENSG00000275765, ENSG00000184350, ENSG00000249456, ENSG00000205155, ENSG00000269656, ENSG00000136149, ENSG00000102931, ENSG00000127774, ENSG00000231327, ENSG00000264107, novel.984, ENSG00000210184, ENSG00000073969, ENSG00000169020, ENSG00000287115, ENSG00000251381, ENSG00000213730, ENSG00000125735, ENSG00000188573, ENSG00000260552, ENSG00000184260, ENSG00000175550, ENSG00000229117, ENSG00000265060, novel.1126, ENSG00000131737, ENSG00000228474, ENSG00000237991, ENSG00000284015, ENSG00000177700, ENSG00000225833, ENSG00000280071, ENSG00000227097, ENSG00000274791, ENSG00000125458, ENSG00000130255, ENSG00000236301, ENSG00000114942, ENSG00000125356, ENSG00000107223, ENSG00000100121, novel.1326, ENSG00000278993, novel.1226, ENSG00000143196, ENSG00000179950, ENSG00000232320, ENSG00000126432, novel.1564, ENSG00000112667, ENSG00000197696, ENSG00000213866, novel.84, ENSG00000235169, ENSG00000234068, novel.1052, ENSG00000240376, novel.717, novel.797, ENSG00000261512, ENSG00000281571, ENSG00000231789, ENSG00000152082, ENSG00000271303, ENSG00000172460, ENSG00000136942, ENSG00000197582, ENSG00000218048, ENSG00000213741, ENSG00000163993, novel.740, novel.843, ENSG00000239264, ENSG00000277459, ENSG00000161970, ENSG00000243829, ENSG00000217716, ENSG00000001630, ENSG00000034510, ENSG00000131116, ENSG00000181524, ENSG00000242071, ENSG00000204922, ENSG00000230266, ENSG00000235618, ENSG00000173457, novel.1435, ENSG00000183011, ENSG00000161016, ENSG00000145494, ENSG00000167664, ENSG00000125995, ENSG00000084674, ENSG00000007080, ENSG00000287331, ENSG00000135940, ENSG00000179168, ENSG00000205078, ENSG00000111678, ENSG00000173272, ENSG00000241494, ENSG00000150779, novel.1554, novel.1057, ENSG00000115255, ENSG00000259072, ENSG00000142484, ENSG00000124733, ENSG00000227081, ENSG00000197766, ENSG00000175701, ENSG00000233198, ENSG00000111775, ENSG00000232838, ENSG00000099795, ENSG00000100938, ENSG00000136867, ENSG00000267795, ENSG00000244187, ENSG00000218426, novel.1345, ENSG00000122133, ENSG00000005075, ENSG00000275494, ENSG00000006327, ENSG00000149806, ENSG00000100142, ENSG00000167193, ENSG00000147804, ENSG00000245112, ENSG00000213218, ENSG00000175756, ENSG00000145592, ENSG00000106268, ENSG00000197956, ENSG00000285458, ENSG00000196683, ENSG00000145912, ENSG00000156482, ENSG00000261472, ENSG00000163815, ENSG00000126603, ENSG00000132329, ENSG00000106123, ENSG00000140264, ENSG00000160285, ENSG00000158716, ENSG00000237506, ENSG00000163344, ENSG00000187808, ENSG00000183617, ENSG00000253683, ENSG00000183876, novel.288, ENSG00000002330, ENSG00000127554, ENSG00000183298, novel.860, ENSG00000074071, ENSG00000197756, ENSG00000254531, ENSG00000198171, ENSG00000214253, ENSG00000278922, ENSG00000215835, ENSG00000051523, ENSG00000166317, novel.796, ENSG00000150750, ENSG00000272523, ENSG00000186493, ENSG00000197540, ENSG00000176340, ENSG00000282988, ENSG00000176845, ENSG00000163220, ENSG00000173156, ENSG00000104979, ENSG00000187051, ENSG00000103266, ENSG00000099800, ENSG00000176046, ENSG00000125652, ENSG00000167799, ENSG00000184508, ENSG00000265246, ENSG00000276529, ENSG00000146677, ENSG00000282221, ENSG00000231500, ENSG00000283154, ENSG00000139714, ENSG00000114473, ENSG00000233762, ENSG00000131495, ENSG00000176101, ENSG00000176973, ENSG00000179967, ENSG00000178093, ENSG00000147485, ENSG00000105640, ENSG00000273253, ENSG00000125878, ENSG00000184730, ENSG00000130545, ENSG00000259032, ENSG00000179085, ENSG00000211897, ENSG00000112245, ENSG00000213326, ENSG00000205220, ENSG00000258429, ENSG00000169100, ENSG00000210135, ENSG00000147889, ENSG00000103152, ENSG00000224877, ENSG00000130829, ENSG00000173113, ENSG00000124701, ENSG00000175416, ENSG00000140743, ENSG00000140988, ENSG00000185614, ENSG00000167536, ENSG00000103254, ENSG00000184897, ENSG00000241563, ENSG00000168040, ENSG00000278845, ENSG00000198258, ENSG00000104140, ENSG00000233276, ENSG00000240087, ENSG00000123144, ENSG00000143314, ENSG00000178449, ENSG00000142534, ENSG00000168701, ENSG00000160256, ENSG00000182899, ENSG00000156875, ENSG00000241468, ENSG00000174307, ENSG00000007255, ENSG00000139239, ENSG00000206177, ENSG00000105404, ENSG00000221821, ENSG00000255262, ENSG00000171858, ENSG00000165502, ENSG00000266274, ENSG00000161677, ENSG00000166595, ENSG00000137309, ENSG00000254719, ENSG00000244313, ENSG00000174917, ENSG00000166924, ENSG00000042493, ENSG00000139197, ENSG00000198937, ENSG00000226287, ENSG00000074842, ENSG00000172780, ENSG00000130725, ENSG00000136425, ENSG00000220842, novel.1558, ENSG00000105373, ENSG00000125901, ENSG00000130748, ENSG00000146540, ENSG00000106244, ENSG00000280649, ENSG00000129911, ENSG00000100097, ENSG00000147586, ENSG00000171222, ENSG00000126267, ENSG00000279692, ENSG00000162849, ENSG00000250220, ENSG00000213442, ENSG00000143793, ENSG00000123395, ENSG00000167136, novel.1143, ENSG00000182154, ENSG00000196544, ENSG00000130312, ENSG00000183128, ENSG00000118181, ENSG00000092841, ENSG00000196976, ENSG00000186007, ENSG00000245848, ENSG00000171223, ENSG00000172428, novel.699, ENSG00000171517, ENSG00000172336, ENSG00000101084, ENSG00000204253, ENSG00000159884, ENSG00000224183, ENSG00000214182, ENSG00000105258, ENSG00000185883, ENSG00000235776, ENSG00000132507, ENSG00000163923, ENSG00000177096, ENSG00000126264, ENSG00000142676, ENSG00000169442, ENSG00000141526, ENSG00000173372, ENSG00000185641, ENSG00000105516, ENSG00000116035, ENSG00000233223, ENSG00000172809, ENSG00000106538, ENSG00000240342, ENSG00000171224, ENSG00000169689, ENSG00000264920, ENSG00000166441, ENSG00000173762, ENSG00000233270, ENSG00000187837, ENSG00000227154, ENSG00000108106, ENSG00000173153, ENSG00000256940, ENSG00000109265, ENSG00000272143, ENSG00000167641, ENSG00000067836, ENSG00000287574, ENSG00000243449, ENSG00000183048, ENSG00000103202, ENSG00000213178, ENSG00000155368, novel.781, ENSG00000121900, ENSG00000189129, ENSG00000103490, ENSG00000134419, ENSG00000111671, ENSG00000160799, ENSG00000154319, ENSG00000171402, ENSG00000237118, ENSG00000183734, ENSG00000055813, ENSG00000172586, ENSG00000134809, novel.235, ENSG00000164967, ENSG00000146066, ENSG00000111639, ENSG00000226085, ENSG00000173267, ENSG00000184227, ENSG00000180015, ENSG00000213862, ENSG00000125971, ENSG00000220472, novel.1487, ENSG00000175602, ENSG00000244734, ENSG00000244716, ENSG00000173660, novel.864, ENSG00000178947, ENSG00000143545, ENSG00000105701, ENSG00000187840, ENSG00000126768, ENSG00000167969, ENSG00000253570, ENSG00000138030, ENSG00000087076, ENSG00000136488, ENSG00000179965, ENSG00000279259, ENSG00000054148, ENSG00000122691, ENSG00000086504, ENSG00000171159, ENSG00000266830, ENSG00000127445, ENSG00000169220, ENSG00000131370, ENSG00000189433, ENSG00000253958, ENSG00000181817, ENSG00000117862, ENSG00000196507, ENSG00000239857, ENSG00000106633, ENSG00000103495, ENSG00000205090, ENSG00000142409, ENSG00000167747, novel.742, ENSG00000136371, ENSG00000166681, ENSG00000130165, ENSG00000143256, ENSG00000128524, ENSG00000269388, ENSG00000274605, ENSG00000165178, ENSG00000133315, ENSG00000212802, ENSG00000112041, ENSG00000166228, ENSG00000185262, ENSG00000103522, ENSG00000172757, ENSG00000239569, ENSG00000129932, ENSG00000148335, ENSG00000285793, ENSG00000162076, ENSG00000119630, ENSG00000258701, ENSG00000185834, novel.353, ENSG00000221869, ENSG00000248121, ENSG00000063854, ENSG00000100605, ENSG00000214309, ENSG00000285634, ENSG00000092758, ENSG00000188186, ENSG00000179403, ENSG00000104964, novel.1499, ENSG00000132967, ENSG00000125534, ENSG00000167996, ENSG00000161281, ENSG00000167526, ENSG00000100379, ENSG00000257315, ENSG00000118640, ENSG00000182379, ENSG00000175906, ENSG00000100365, ENSG00000130208, ENSG00000217130, ENSG00000174109, ENSG00000229809, ENSG00000213144, ENSG00000105327, ENSG00000055070, ENSG00000130811, ENSG00000142541, ENSG00000251271, ENSG00000187796, ENSG00000167642, ENSG00000198356, ENSG00000160948, ENSG00000203724, ENSG00000203760, ENSG00000104897, ENSG00000008735, ENSG00000278869, ENSG00000197958, ENSG00000225285, ENSG00000141934, ENSG00000224870, ENSG00000179271, ENSG00000128185, ENSG00000141639, ENSG00000130005, ENSG00000165912, ENSG00000130222, ENSG00000198918, ENSG00000214192, ENSG00000106367, ENSG00000134825, ENSG00000175573, ENSG00000119013, ENSG00000130520, ENSG00000173013, ENSG00000090238, ENSG00000106211, ENSG00000180596, ENSG00000090013, ENSG00000175445, ENSG00000158828, ENSG00000007520, ENSG00000023191, ENSG00000115350, novel.928, ENSG00000179431, ENSG00000135392, ENSG00000099203, ENSG00000215845, ENSG00000213563, ENSG00000099804, ENSG00000167157, ENSG00000137970, ENSG00000100122, ENSG00000167244, ENSG00000186603, ENSG00000127184, novel.1023, ENSG00000171421, ENSG00000105559, ENSG00000100804, ENSG00000063177, ENSG00000173227, ENSG00000139410, ENSG00000186998, ENSG00000101460, ENSG00000184840, ENSG00000198915, ENSG00000105193, ENSG00000129152, ENSG00000285976, ENSG00000198680, ENSG00000170469, ENSG00000089486, ENSG00000131143, ENSG00000102309, ENSG00000184281, ENSG00000114391, ENSG00000154102, ENSG00000169583, ENSG00000015285, ENSG00000175315, ENSG00000109919, ENSG00000107317, novel.845, ENSG00000230330, ENSG00000051596, ENSG00000227063, ENSG00000069188, ENSG00000006015, ENSG00000064961, ENSG00000148803, ENSG00000171443, ENSG00000269313, novel.649, ENSG00000260260, ENSG00000130203, ENSG00000205138, ENSG00000121552, ENSG00000139675, ENSG00000072506, ENSG00000167513, ENSG00000164587, ENSG00000106803, ENSG00000232573, ENSG00000099860, ENSG00000176894, ENSG00000196465, ENSG00000099849, ENSG00000181588, ENSG00000267390, ENSG00000125970, ENSG00000167671, ENSG00000213465, ENSG00000228589, ENSG00000162244, ENSG00000168061, ENSG00000085265, ENSG00000225259, ENSG00000197258, ENSG00000142235, ENSG00000233954, ENSG00000178896, ENSG00000148334, ENSG00000105519, ENSG00000228253, ENSG00000174015, ENSG00000231351, ENSG00000123080, ENSG00000143546, ENSG00000133134, ENSG00000130707, ENSG00000259705, ENSG00000160446, ENSG00000177283, ENSG00000135404, ENSG00000130204, ENSG00000162882, ENSG00000060558, ENSG00000177600, ENSG00000130382, ENSG00000114631, ENSG00000186577, ENSG00000126709, ENSG00000140564, ENSG00000167515, ENSG00000130332, ENSG00000100056, ENSG00000177954, ENSG00000236552, ENSG00000179632, ENSG00000140990, ENSG00000100399, ENSG00000144713, ENSG00000128805, novel.921, ENSG00000085117, ENSG00000123353, ENSG00000104883, ENSG00000197620, ENSG00000220749, ENSG00000112559, ENSG00000164405, ENSG00000241553, ENSG00000103363, ENSG00000174744, ENSG00000198832, ENSG00000185298, ENSG00000051128, ENSG00000163956, ENSG00000119986, ENSG00000159761, ENSG00000049089, ENSG00000175634, ENSG00000115266, ENSG00000226243, ENSG00000234287, ENSG00000148303, ENSG00000125520, ENSG00000103260, ENSG00000198355, ENSG00000099817, ENSG00000198931, ENSG00000153395, ENSG00000183822, ENSG00000179588, ENSG00000114115, ENSG00000117154, novel.857, ENSG00000184363, ENSG00000164713, ENSG00000163170, ENSG00000198736, ENSG00000149761, ENSG00000187244, ENSG00000087088, ENSG00000158715, ENSG00000270504, ENSG00000244398, ENSG00000172057, ENSG00000198242, ENSG00000129235, ENSG00000168101, ENSG00000184076, ENSG00000108298, ENSG00000182512, ENSG00000165171, ENSG00000144741, ENSG00000006118, ENSG00000130731, ENSG00000196878, ENSG00000116288, ENSG00000100075, ENSG00000095970, ENSG00000132881, ENSG00000186468, ENSG00000167037, ENSG00000105722, ENSG00000168899, ENSG00000162300, ENSG00000126062, ENSG00000167543, ENSG00000160570, ENSG00000196196, novel.927, ENSG00000090273, ENSG00000100246, ENSG00000114854, ENSG00000213213, ENSG00000149273, ENSG00000125910, ENSG00000153048, ENSG00000118804, ENSG00000139438, ENSG00000065057, ENSG00000084207, ENSG00000104886, ENSG00000123159, ENSG00000105401, ENSG00000164182, novel.933, ENSG00000198467, ENSG00000240036, ENSG00000067606, ENSG00000136490, ENSG00000235098, ENSG00000149929, ENSG00000237149, ENSG00000116586, ENSG00000126458, ENSG00000141522, ENSG00000131469, ENSG00000280828, ENSG00000184481, ENSG00000228300, ENSG00000189306, ENSG00000227473, ENSG00000169564, ENSG00000105254, ENSG00000105963, ENSG00000065268, ENSG00000108961, ENSG00000125656, ENSG00000042753, ENSG00000120071, ENSG00000185347, ENSG00000141965, ENSG00000008382, ENSG00000211450, ENSG00000107404, ENSG00000083845, ENSG00000182117, ENSG00000161888, ENSG00000127220, ENSG00000233927, ENSG00000204237, ENSG00000275074, ENSG00000263266, ENSG00000185633, ENSG00000053371, ENSG00000066336, ENSG00000198546, ENSG00000242372, ENSG00000196758, ENSG00000131401, ENSG00000278970, ENSG00000117691, ENSG00000278921, ENSG00000160813, ENSG00000126218, ENSG00000164897, ENSG00000160877, ENSG00000149418, ENSG00000169217, novel.777, ENSG00000116871, ENSG00000142634, ENSG00000105321, ENSG00000112695, ENSG00000124074, ENSG00000260549, ENSG00000182575, ENSG00000141933, ENSG00000161671, ENSG00000159335, ENSG00000185340, ENSG00000233830, ENSG00000103145, ENSG00000169976, ENSG00000173369, ENSG00000176619, ENSG00000107282, ENSG00000126767, ENSG00000105374, ENSG00000175467, ENSG00000184209, ENSG00000065978, ENSG00000161999, ENSG00000169692, ENSG00000102103, ENSG00000226137, ENSG00000232533, ENSG00000124172, ENSG00000090971, ENSG00000176058, ENSG00000167700, ENSG00000160685, ENSG00000102003, ENSG00000230513, ENSG00000237004, ENSG00000183196, ENSG00000153774, ENSG00000095906, ENSG00000256073, ENSG00000197744, ENSG00000142459, ENSG00000243199, ENSG00000159189, ENSG00000173141, ENSG00000126254, ENSG00000129538, ENSG00000101400, ENSG00000198892, ENSG00000158747, ENSG00000119421, ENSG00000091704, ENSG00000161653, ENSG00000167645, ENSG00000122386, ENSG00000060491, ENSG00000161920, ENSG00000011600, novel.1125, ENSG00000174028, ENSG00000125746, ENSG00000115129, ENSG00000107874, ENSG00000108107, ENSG00000135924, ENSG00000025708, ENSG00000198517, ENSG00000119705, ENSG00000168884, ENSG00000248905, ENSG00000105364, ENSG00000141985, ENSG00000259642, ENSG00000205744, ENSG00000139405, ENSG00000183684, ENSG00000226221, ENSG00000162551, ENSG00000188483, ENSG00000245060, ENSG00000197451, novel.811, ENSG00000169228, ENSG00000099385, ENSG00000154518, ENSG00000213261, ENSG00000121653, ENSG00000136830, ENSG00000286190, ENSG00000114383, ENSG00000131408, ENSG00000161981, ENSG00000170619, novel.1123, ENSG00000159685, ENSG00000091640, ENSG00000244280, ENSG00000221983, ENSG00000123349, ENSG00000179862, ENSG00000141552, ENSG00000198003, ENSG00000287104, ENSG00000230202, ENSG00000179743, ENSG00000100216, ENSG00000172216, ENSG00000197019, ENSG00000102030, ENSG00000158042, ENSG00000166289, ENSG00000156603, ENSG00000131446, ENSG00000099869, ENSG00000064490, ENSG00000246705, ENSG00000161642, ENSG00000143575, ENSG00000090581, ENSG00000110492, ENSG00000182809, ENSG00000130810, ENSG00000237214, ENSG00000277702, ENSG00000070729, ENSG00000162191, ENSG00000197180, ENSG00000105699, ENSG00000071082, ENSG00000185453, ENSG00000116670, ENSG00000232389, ENSG00000129925, ENSG00000107833, ENSG00000106266, novel.788, ENSG00000188191, ENSG00000100836, ENSG00000178096, ENSG00000158526, ENSG00000104679, ENSG00000116039, ENSG00000155622, ENSG00000143774, ENSG00000115325, ENSG00000156381, ENSG00000183309, ENSG00000218052, ENSG00000071655, ENSG00000125743, ENSG00000073050, ENSG00000182768, ENSG00000103024, ENSG00000198835, ENSG00000134463, ENSG00000162302, ENSG00000125753, ENSG00000105048, ENSG00000226415, ENSG00000221990, ENSG00000197483, ENSG00000167397, ENSG00000126460, ENSG00000139725, ENSG00000099330, ENSG00000160993, ENSG00000160221, ENSG00000160113, ENSG00000198840, ENSG00000149923, ENSG00000155366, ENSG00000103274, ENSG00000141744, ENSG00000169683, ENSG00000117122, ENSG00000181404, ENSG00000181274, ENSG00000064932, ENSG00000173581, ENSG00000174886, ENSG00000100162, ENSG00000177697, ENSG00000007376, ENSG00000100300, ENSG00000168329, ENSG00000143553, ENSG00000143862, ENSG00000133275, ENSG00000142937, ENSG00000144583, ENSG00000172366, ENSG00000254858, ENSG00000247596, ENSG00000174547, ENSG00000168894, ENSG00000116717, novel.1118, ENSG00000131944, ENSG00000179091, ENSG00000169223, ENSG00000111786, ENSG00000270170, ENSG00000127837, ENSG00000100842, ENSG00000123064, ENSG00000264281, ENSG00000159069, ENSG00000100294, ENSG00000228889, ENSG00000170667, ENSG00000164081, ENSG00000188820, ENSG00000139192, ENSG00000129968, ENSG00000225178, ENSG00000180879, ENSG00000238227, ENSG00000170632, ENSG00000119559, ENSG00000001460, ENSG00000135047, ENSG00000170837, ENSG00000133142, ENSG00000136732, ENSG00000180875, ENSG00000166557, ENSG00000159128, ENSG00000130734, ENSG00000198324, ENSG00000139112, ENSG00000224307, ENSG00000163517, ENSG00000175334, novel.1107, ENSG00000166166, ENSG00000177830, ENSG00000275234, ENSG00000148450, ENSG00000130159, ENSG00000103187, ENSG00000100319, ENSG00000179041, ENSG00000150977, ENSG00000185909, ENSG00000122121, ENSG00000171853, ENSG00000161955, ENSG00000197903, ENSG00000130522, ENSG00000143816, ENSG00000100949, ENSG00000174276, ENSG00000189343, ENSG00000137161, ENSG00000130717, ENSG00000197982, ENSG00000197562, ENSG00000099341, ENSG00000019582, ENSG00000269386, ENSG00000130706, ENSG00000167680, ENSG00000267296, ENSG00000168005, ENSG00000161179, ENSG00000110195, ENSG00000122971, ENSG00000162910, ENSG00000102879, ENSG00000258920, ENSG00000121281, novel.1222, ENSG00000099992, novel.66, ENSG00000110711, ENSG00000110717, ENSG00000103549, ENSG00000130669, ENSG00000126453, ENSG00000063245, ENSG00000174996, ENSG00000165507, ENSG00000260236, ENSG00000185338, ENSG00000141741, ENSG00000117984, ENSG00000037241, ENSG00000235568, ENSG00000172725, ENSG00000124466, ENSG00000162931, ENSG00000105248, ENSG00000235043, ENSG00000179772, ENSG00000168496, ENSG00000179348, ENSG00000118046, ENSG00000131669, ENSG00000128228, ENSG00000108774, ENSG00000159840, ENSG00000109390, ENSG00000178718, ENSG00000142168, ENSG00000105355, ENSG00000165879, ENSG00000116863, ENSG00000101951, ENSG00000255135, ENSG00000167674, ENSG00000174851, ENSG00000273619, ENSG00000110080, ENSG00000174021, ENSG00000135631, ENSG00000215861, ENSG00000124702, ENSG00000123989, ENSG00000125611, ENSG00000141577, ENSG00000169612, ENSG00000236439, ENSG00000232346, ENSG00000284753, ENSG00000076864, ENSG00000166592, ENSG00000173992, ENSG00000168071, ENSG00000167779, ENSG00000185112, ENSG00000110047, ENSG00000189409, ENSG00000171700, ENSG00000135736, ENSG00000157911, ENSG00000185813, ENSG00000102119, ENSG00000177191, ENSG00000178605, ENSG00000180992, ENSG00000136888, ENSG00000004059, ENSG00000135441, ENSG00000087884, ENSG00000156467, ENSG00000122490, ENSG00000105711, ENSG00000186834, ENSG00000115286, ENSG00000198746, ENSG00000104856, ENSG00000178980, ENSG00000126461, ENSG00000133316, ENSG00000101057, ENSG00000224578, ENSG00000101160, ENSG00000161243, ENSG00000184436, ENSG00000135363, ENSG00000197093, ENSG00000166002, ENSG00000168875, ENSG00000111432, ENSG00000179277, ENSG00000130755, ENSG00000179115, ENSG00000257704, ENSG00000085998, novel.691, ENSG00000168209, ENSG00000188290, ENSG00000275464, novel.743, ENSG00000130733, ENSG00000173465, ENSG00000134597, ENSG00000172663, ENSG00000167658, ENSG00000129250, ENSG00000168060, ENSG00000224051, ENSG00000121769, ENSG00000172301, ENSG00000196502, ENSG00000103245, ENSG00000117410, ENSG00000223799, ENSG00000165917, ENSG00000198755, ENSG00000163382, ENSG00000147443, ENSG00000166886, ENSG00000130724, ENSG00000107738, ENSG00000223573, ENSG00000116990, ENSG00000161914, ENSG00000178826, ENSG00000198960, ENSG00000233016, ENSG00000112576, ENSG00000154342, ENSG00000233937, ENSG00000156860, ENSG00000175854, ENSG00000171388, ENSG00000135506, ENSG00000117308, ENSG00000101246, ENSG00000164051, ENSG00000125651, ENSG00000123810, ENSG00000178860, ENSG00000273117, ENSG00000169230, ENSG00000104872, ENSG00000139832, ENSG00000147403, ENSG00000167965, ENSG00000167994, ENSG00000176978, ENSG00000212664, ENSG00000127922, ENSG00000105698, ENSG00000173207, ENSG00000171219, ENSG00000142279, ENSG00000142910, ENSG00000135617, ENSG00000142507, ENSG00000253313, ENSG00000108819, ENSG00000128563, ENSG00000109610, ENSG00000079313, ENSG00000178741, ENSG00000102996, ENSG00000108518, ENSG00000173171, ENSG00000116663, ENSG00000142546, ENSG00000105379, ENSG00000112561, ENSG00000165804, ENSG00000171813, ENSG00000169957, ENSG00000173540, ENSG00000236801, ENSG00000163870, ENSG00000177380, ENSG00000182208, ENSG00000147123, ENSG00000167100, ENSG00000212864, ENSG00000130244, ENSG00000165233, ENSG00000186395, ENSG00000159423, ENSG00000169372, ENSG00000172818, ENSG00000104213, ENSG00000256628, ENSG00000119333, ENSG00000089248, ENSG00000008283, ENSG00000234797, ENSG00000089685, ENSG00000196411, ENSG00000274286, ENSG00000110536, ENSG00000082515, ENSG00000178057, ENSG00000184162, ENSG00000169750, ENSG00000106153, ENSG00000177469, ENSG00000141994, ENSG00000133597, ENSG00000170779, ENSG00000164889, ENSG00000242299, ENSG00000182871, ENSG00000239672, ENSG00000130175, ENSG00000119673, ENSG00000180155, novel.696, ENSG00000214530, ENSG00000100292, ENSG00000090447, ENSG00000183397, ENSG00000157881, ENSG00000165724, ENSG00000050820, ENSG00000125505, ENSG00000154764, ENSG00000133216, ENSG00000161682, ENSG00000181396, ENSG00000170473, ENSG00000170421, ENSG00000184207, ENSG00000167468, ENSG00000119714, ENSG00000116774, ENSG00000129355, ENSG00000105227, ENSG00000213462, ENSG00000123999, ENSG00000070731, ENSG00000106665, ENSG00000186212, ENSG00000153093, ENSG00000204673, ENSG00000106330, ENSG00000261221, ENSG00000146670, ENSG00000151729, ENSG00000160783, ENSG00000089220, ENSG00000196642, ENSG00000189171, ENSG00000099624, ENSG00000167797, ENSG00000105372, ENSG00000134222, ENSG00000179526, ENSG00000177374, ENSG00000184924, ENSG00000129245, ENSG00000164626, ENSG00000166997, ENSG00000130695, ENSG00000121933, ENSG00000177868, ENSG00000125744, ENSG00000013306, ENSG00000259431, ENSG00000121680, ENSG00000142544, ENSG00000174080, ENSG00000183207, ENSG00000078808, ENSG00000198055, ENSG00000130511, ENSG00000110400, ENSG00000197249, ENSG00000055950, ENSG00000105669, ENSG00000088899, ENSG00000166337, ENSG00000140832, ENSG00000186827, ENSG00000225614, ENSG00000166428, ENSG00000026559, ENSG00000125352, ENSG00000257103, ENSG00000163346, ENSG00000099875, ENSG00000170604, ENSG00000270885, ENSG00000133243, ENSG00000106689, ENSG00000230897, ENSG00000170955, ENSG00000196205, ENSG00000122952, ENSG00000108479, ENSG00000143382, ENSG00000105298, ENSG00000076826, ENSG00000138495, ENSG00000183779, ENSG00000119383, ENSG00000166562, ENSG00000118960, ENSG00000160213, ENSG00000126464, ENSG00000178951, ENSG00000104783, ENSG00000237190, ENSG00000168569, ENSG00000130311, ENSG00000219607, ENSG00000137288, ENSG00000076984, ENSG00000105229, ENSG00000176788, ENSG00000229833, ENSG00000128283, ENSG00000172354, ENSG00000135446, ENSG00000169515, ENSG00000011009, ENSG00000148926, ENSG00000161091, ENSG00000070423, ENSG00000184113, ENSG00000110200, ENSG00000063244, ENSG00000188613, ENSG00000162066, ENSG00000100316, ENSG00000189060, ENSG00000196182, ENSG00000090097, ENSG00000105472, ENSG00000197858, ENSG00000120913, ENSG00000141504, ENSG00000177556, ENSG00000175061, ENSG00000166452, ENSG00000262919, ENSG00000173327, ENSG00000125817, ENSG00000163795, ENSG00000133985, ENSG00000108523, ENSG00000166133, ENSG00000162576, ENSG00000182218, ENSG00000189043, ENSG00000077463, ENSG00000188112, ENSG00000175063, ENSG00000168653, ENSG00000119431, ENSG00000197021, ENSG00000267317, ENSG00000149925, ENSG00000107816, ENSG00000096080, ENSG00000101439, ENSG00000275964, ENSG00000105671, ENSG00000120725, ENSG00000228672, ENSG00000259956, ENSG00000101412, ENSG00000136286, ENSG00000175707, ENSG00000234851, ENSG00000100299, ENSG00000198816, ENSG00000100417, ENSG00000101365, ENSG00000168734, ENSG00000139998, ENSG00000100823, ENSG00000088356, ENSG00000184990, ENSG00000099901, ENSG00000146063, ENSG00000172270, ENSG00000214756, ENSG00000249565, ENSG00000127540, ENSG00000117411, ENSG00000134460, ENSG00000107140, ENSG00000138172, ENSG00000259863, ENSG00000109475, ENSG00000171530, ENSG00000165905, ENSG00000141574, ENSG00000101335, ENSG00000188846, ENSG00000125691, ENSG00000169738, ENSG00000125898, ENSG00000213888, ENSG00000108528, ENSG00000130701, ENSG00000068394, ENSG00000188486, ENSG00000188042, ENSG00000160214, ENSG00000161956, ENSG00000148908, ENSG00000120896, ENSG00000215021, ENSG00000177045, ENSG00000077348, ENSG00000163132, ENSG00000137193, ENSG00000165272, ENSG00000224831, ENSG00000142655, ENSG00000172531, ENSG00000143977, ENSG00000099364, ENSG00000102871, ENSG00000110876, ENSG00000274213, ENSG00000126467, ENSG00000100997, ENSG00000138606, ENSG00000162522, ENSG00000159377, ENSG00000176410, ENSG00000130300, ENSG00000161513, ENSG00000204947, ENSG00000136720, ENSG00000139637, ENSG00000149115, ENSG00000142669, ENSG00000130758, ENSG00000126088, ENSG00000162729, ENSG00000171992, ENSG00000184216, ENSG00000141959, ENSG00000106628, ENSG00000196365, ENSG00000166189, ENSG00000183784, ENSG00000137154, ENSG00000171823, novel.859, ENSG00000127191, ENSG00000128274, ENSG00000125835, ENSG00000007047, ENSG00000041988, ENSG00000063241, ENSG00000138111, ENSG00000283041, ENSG00000167703, ENSG00000185361, ENSG00000175193, novel.1552, ENSG00000102178, ENSG00000149823, ENSG00000247077, ENSG00000181610, ENSG00000116857, ENSG00000170315, ENSG00000090382, ENSG00000167815, ENSG00000016391, ENSG00000254622, ENSG00000126903, ENSG00000169635, ENSG00000167862, ENSG00000143543, ENSG00000064655, ENSG00000256053, ENSG00000114650, ENSG00000108786, ENSG00000107262, ENSG00000168476, ENSG00000141582, ENSG00000198842, ENSG00000104983, ENSG00000170468, ENSG00000164919, ENSG00000165886, ENSG00000128272, ENSG00000249353, ENSG00000153443, ENSG00000214223, ENSG00000111669, ENSG00000186174, ENSG00000159352, ENSG00000185043, ENSG00000147684, ENSG00000104866, ENSG00000136840, ENSG00000090266, ENSG00000161036, ENSG00000276600, ENSG00000197457, ENSG00000114956, ENSG00000104915, ENSG00000149792, ENSG00000143321, ENSG00000168077, ENSG00000184752, ENSG00000171552, ENSG00000173264, ENSG00000288398, ENSG00000087074, ENSG00000250317, ENSG00000136717, ENSG00000179627, ENSG00000160014, ENSG00000155066, ENSG00000182487, ENSG00000161277, ENSG00000143420, ENSG00000164104, ENSG00000105223, ENSG00000196700, ENSG00000124839, ENSG00000167770, ENSG00000176532, ENSG00000176108, ENSG00000087086, ENSG00000274180, ENSG00000103485, ENSG00000120457, ENSG00000081692, ENSG00000180891, ENSG00000100591, ENSG00000170638, ENSG00000181991, ENSG00000198858, ENSG00000128463, ENSG00000170439, ENSG00000114767, ENSG00000145050, ENSG00000079999, ENSG00000071859, ENSG00000108798, ENSG00000100106, ENSG00000149541, ENSG00000071246, ENSG00000189143, ENSG00000184208, ENSG00000162073, ENSG00000198420, ENSG00000173926, ENSG00000144868, ENSG00000241878, ENSG00000134248, ENSG00000105518, ENSG00000171425, ENSG00000176170, ENSG00000168028, ENSG00000162385, ENSG00000166925, ENSG00000101444, ENSG00000259917, ENSG00000179943, ENSG00000177854, ENSG00000164087, ENSG00000167395, ENSG00000115268, ENSG00000213015, ENSG00000178952, ENSG00000154723, ENSG00000174748, ENSG00000110011, ENSG00000176485, ENSG00000107902, ENSG00000167962, ENSG00000113811, ENSG00000159176, ENSG00000213740, ENSG00000108344, ENSG00000115275, ENSG00000123892, ENSG00000105289, ENSG00000116809, ENSG00000163191, ENSG00000185236, ENSG00000131475, ENSG00000141480, ENSG00000160818, ENSG00000232472, ENSG00000183648, ENSG00000088726, ENSG00000165916, ENSG00000004975, ENSG00000100243, ENSG00000162817, ENSG00000040487, ENSG00000213553, ENSG00000188986, ENSG00000088256, ENSG00000267858, ENSG00000183018, ENSG00000141736, ENSG00000125503, ENSG00000173918, ENSG00000198624, ENSG00000168002, ENSG00000174903, ENSG00000136710, ENSG00000136059, ENSG00000132471, ENSG00000165644, ENSG00000129103, ENSG00000183828, ENSG00000177548, ENSG00000173511, ENSG00000137818, ENSG00000000938, ENSG00000105677, ENSG00000125445, ENSG00000135916, ENSG00000064195, ENSG00000160917, ENSG00000171298, ENSG00000166016, ENSG00000005238, ENSG00000105135, ENSG00000067221, ENSG00000177106, ENSG00000049283, ENSG00000254470, ENSG00000103496, ENSG00000124243, ENSG00000008988, ENSG00000188612, ENSG00000161395, ENSG00000131981, ENSG00000177732, ENSG00000156127, ENSG00000165475, ENSG00000177733, ENSG00000033100, ENSG00000087152, ENSG00000065054, ENSG00000160688, ENSG00000143198, ENSG00000213347, ENSG00000123933, ENSG00000168994, ENSG00000108826, ENSG00000083838, ENSG00000115041, ENSG00000273472, ENSG00000131242, ENSG00000167110, ENSG00000224546, ENSG00000162998, ENSG00000123815, ENSG00000129354, ENSG00000138867, ENSG00000161847, ENSG00000171863, ENSG00000177030, ENSG00000171169, ENSG00000184557, ENSG00000126934, ENSG00000125731, ENSG00000167740, ENSG00000089639, ENSG00000099864, ENSG00000175324, ENSG00000187147, ENSG00000176092, ENSG00000105058, ENSG00000111716, ENSG00000215440, ENSG00000072518, ENSG00000043039, ENSG00000126012, ENSG00000131153, ENSG00000100284, ENSG00000107954, ENSG00000079616, ENSG00000242485, ENSG00000169084, ENSG00000110700, ENSG00000087077, ENSG00000132661, ENSG00000184602, ENSG00000163832, ENSG00000168090, ENSG00000025770, ENSG00000272841, ENSG00000159714, ENSG00000139546, ENSG00000100865, ENSG00000160972, ENSG00000184470, ENSG00000176182, ENSG00000125730, ENSG00000105755, ENSG00000101220, ENSG00000182400, ENSG00000112306, ENSG00000060656, ENSG00000185187, ENSG00000142186, ENSG00000173442, ENSG00000172831, ENSG00000071889, ENSG00000197043, ENSG00000141968, ENSG00000176946, ENSG00000179262, ENSG00000116017, ENSG00000107338, ENSG00000149357, ENSG00000125148, ENSG00000198873, ENSG00000188026, ENSG00000177352, novel.1196, ENSG00000160226, ENSG00000136718, ENSG00000180198, ENSG00000135390, ENSG00000106683, ENSG00000116096, ENSG00000170545, ENSG00000132470, ENSG00000100413, ENSG00000137100, ENSG00000114902, ENSG00000060971, ENSG00000132612, ENSG00000034152, ENSG00000115363, ENSG00000160789, ENSG00000132517, ENSG00000167670, ENSG00000080189, ENSG00000106400, ENSG00000145945, ENSG00000141101, ENSG00000137198, ENSG00000161714, ENSG00000109103, ENSG00000182095, ENSG00000110697, ENSG00000136463, ENSG00000137166, ENSG00000196453, ENSG00000149782, ENSG00000133265, ENSG00000054116, ENSG00000101182, ENSG00000117407, ENSG00000213300, ENSG00000105341, ENSG00000137106, ENSG00000168303, ENSG00000104805, ENSG00000171160, ENSG00000167930, ENSG00000129158, ENSG00000239779, ENSG00000171953, ENSG00000142444, ENSG00000088833, ENSG00000164978, ENSG00000136295, ENSG00000147144, ENSG00000264364, ENSG00000109854, ENSG00000286532, ENSG00000131055, ENSG00000100764, ENSG00000156711, ENSG00000130479, ENSG00000168398, ENSG00000157216, ENSG00000115944, ENSG00000145335, ENSG00000121766, ENSG00000030110, ENSG00000159228, ENSG00000130313, ENSG00000125843, ENSG00000156411, ENSG00000163485, |
|  | up | ENSG00000135679, ENSG00000214135, ENSG00000164741, ENSG00000114861, ENSG00000003756, ENSG00000113319, ENSG00000162999, ENSG00000120798, ENSG00000180376, ENSG00000197603, ENSG00000272316, ENSG00000196911, ENSG00000225855, ENSG00000260941, ENSG00000158711, ENSG00000138346, ENSG00000163629, ENSG00000111879, ENSG00000122008, ENSG00000144935, ENSG00000137449, ENSG00000189050, ENSG00000115904, ENSG00000168566, ENSG00000197776, ENSG00000109618, ENSG00000137672, ENSG00000180182, ENSG00000180479, ENSG00000139292, ENSG00000158987, ENSG00000157741, ENSG00000149311, ENSG00000133710, ENSG00000214413, ENSG00000115524, ENSG00000119285, ENSG00000106692, ENSG00000172167, ENSG00000155903, ENSG00000107201, ENSG00000076641, ENSG00000115421, ENSG00000188171, ENSG00000102755, ENSG00000166349, ENSG00000136603, ENSG00000270049, ENSG00000198734, ENSG00000132485, ENSG00000264538, ENSG00000223959, ENSG00000245849, ENSG00000138380, ENSG00000150907, ENSG00000164188, ENSG00000157036, novel.338, ENSG00000100890, ENSG00000126804, ENSG00000117616, ENSG00000120693, ENSG00000163714, ENSG00000197608, ENSG00000138050, ENSG00000134352, ENSG00000181450, ENSG00000227060, ENSG00000243943, ENSG00000158079, ENSG00000118596, ENSG00000138735, ENSG00000165868, ENSG00000064933, ENSG00000197969, ENSG00000256525, ENSG00000018189, ENSG00000137871, novel.6, ENSG00000182700, ENSG00000164125, novel.1495, ENSG00000164074, ENSG00000226648, ENSG00000136160, ENSG00000198155, ENSG00000175054, ENSG00000279159, ENSG00000124193, ENSG00000139289, ENSG00000174799, ENSG00000235823, ENSG00000114770, ENSG00000168939, ENSG00000163637, ENSG00000144182, ENSG00000186260, ENSG00000166479, ENSG00000010244, ENSG00000075213, ENSG00000080298, ENSG00000144559, ENSG00000106804, ENSG00000189195, ENSG00000198466, ENSG00000204147, ENSG00000154645, ENSG00000117620, ENSG00000007062, ENSG00000204116, ENSG00000133858, ENSG00000129534, ENSG00000173611, ENSG00000167555, ENSG00000129317, ENSG00000082175, ENSG00000235703, ENSG00000120458, ENSG00000182903, ENSG00000169629, ENSG00000120784, ENSG00000026103, ENSG00000186814, ENSG00000118965, ENSG00000214021, ENSG00000198743, ENSG00000203880, ENSG00000047188, ENSG00000158966, ENSG00000152760, novel.452, ENSG00000171817, ENSG00000249464, ENSG00000184445, ENSG00000238113, ENSG00000174718, novel.1135, ENSG00000204130, ENSG00000227502, ENSG00000125814, ENSG00000197372, ENSG00000053900, ENSG00000269929, ENSG00000144445, ENSG00000130844, ENSG00000223546, ENSG00000198075, ENSG00000273142, ENSG00000198513, ENSG00000203867, ENSG00000215386, ENSG00000116754, ENSG00000233006, ENSG00000257354, ENSG00000162614, ENSG00000282851, ENSG00000177853, ENSG00000160961, ENSG00000107951, ENSG00000108848, ENSG00000156787, ENSG00000115355, ENSG00000155158, ENSG00000132274, ENSG00000269825, ENSG00000185246, ENSG00000110852, ENSG00000149346, ENSG00000143995, ENSG00000137501, ENSG00000231731, ENSG00000164114, ENSG00000162971, ENSG00000234456, ENSG00000214548, ENSG00000163596, ENSG00000117600, ENSG00000169432, ENSG00000269842, ENSG00000088756, ENSG00000134987, ENSG00000124406, ENSG00000172748, ENSG00000161547, ENSG00000164764, ENSG00000117228, ENSG00000019991, ENSG00000247679, ENSG00000047634, ENSG00000229358, ENSG00000137831, ENSG00000179406, ENSG00000139974, ENSG00000280347, ENSG00000106258, ENSG00000171016, ENSG00000262370, ENSG00000286584, ENSG00000068885, ENSG00000198774, ENSG00000197601, ENSG00000196865, ENSG00000160062, ENSG00000197056, ENSG00000279041, novel.591, ENSG00000215769, ENSG00000171444, ENSG00000214765, novel.552, ENSG00000153933, ENSG00000254996, ENSG00000114423, ENSG00000089775, ENSG00000245958, ENSG00000056277, ENSG00000279118, ENSG00000163867, ENSG00000213777, ENSG00000196705, novel.149, ENSG00000162929, ENSG00000153914, ENSG00000169826, ENSG00000143127, ENSG00000118495, ENSG00000005801, ENSG00000156103, ENSG00000003987, ENSG00000140285, ENSG00000164066, ENSG00000167371, ENSG00000132204, ENSG00000163738, ENSG00000198046, ENSG00000168772, ENSG00000251595, ENSG00000188227, ENSG00000152926, ENSG00000278768, ENSG00000013588, ENSG00000109046, ENSG00000156265, ENSG00000130684, ENSG00000256087, ENSG00000269821, ENSG00000149231, ENSG00000143178, ENSG00000263272, ENSG00000113448, ENSG00000059588, ENSG00000144802, ENSG00000063127, ENSG00000164309, ENSG00000285669, ENSG00000101333, ENSG00000147180, ENSG00000275764, ENSG00000135315, ENSG00000187790, ENSG00000120051, ENSG00000256223, ENSG00000138615, ENSG00000135063, ENSG00000251095, ENSG00000270804, ENSG00000224934, ENSG00000118922, ENSG00000254535, ENSG00000129003, ENSG00000117569, ENSG00000225891, ENSG00000269235, ENSG00000162769, ENSG00000147118, ENSG00000104938, ENSG00000279765, ENSG00000165338, ENSG00000272180, ENSG00000163617, ENSG00000100479, ENSG00000249669, ENSG00000234420, ENSG00000259673, ENSG00000172244, ENSG00000109452, ENSG00000119707, novel.900, ENSG00000124224, ENSG00000272645, ENSG00000279277, ENSG00000237298, ENSG00000281103, ENSG00000260855, ENSG00000123870, ENSG00000238197, ENSG00000132424, ENSG00000151849, ENSG00000136147, ENSG00000127241, ENSG00000151835, ENSG00000231160, ENSG00000250602, ENSG00000267934, ENSG00000143702, ENSG00000204311, ENSG00000156042, ENSG00000064651, ENSG00000102780, ENSG00000196268, ENSG00000272758, ENSG00000167220, ENSG00000113240, ENSG00000145908, ENSG00000172766, ENSG00000154265, ENSG00000271335, ENSG00000111335, ENSG00000122483, ENSG00000262001, ENSG00000153956, ENSG00000186020, ENSG00000283050, ENSG00000287837, ENSG00000033867, ENSG00000257511, ENSG00000268362, ENSG00000234494, ENSG00000229404, ENSG00000260317, ENSG00000272356, ENSG00000258890, ENSG00000251136, ENSG00000196329, ENSG00000151967, ENSG00000121454, ENSG00000239665, ENSG00000144619, ENSG00000282943, ENSG00000264456, ENSG00000110318, ENSG00000279738, ENSG00000182983, ENSG00000144642, ENSG00000197124, ENSG00000261786, ENSG00000181104, ENSG00000147162, novel.897, ENSG00000151687, ENSG00000165029, ENSG00000082196, ENSG00000115604, ENSG00000198040, ENSG00000213066, ENSG00000166928, ENSG00000271270, ENSG00000115419, novel.1577, ENSG00000055732, ENSG00000175893, ENSG00000243970, ENSG00000286379, ENSG00000172243, ENSG00000168386, ENSG00000135870, ENSG00000261490, ENSG00000154874, ENSG00000180532, ENSG00000131127, ENSG00000255910, ENSG00000223745, ENSG00000185986, ENSG00000206573, ENSG00000101751, ENSG00000176293, ENSG00000109771, novel.360, ENSG00000196247, ENSG00000167524, ENSG00000274536, ENSG00000251022, ENSG00000249307, ENSG00000253636, ENSG00000159086, ENSG00000074935, ENSG00000122435, ENSG00000180785, ENSG00000137872, ENSG00000180953, ENSG00000231721, ENSG00000102287, ENSG00000197594, novel.271, ENSG00000276517, ENSG00000198865, ENSG00000180287, ENSG00000166801, ENSG00000248429, ENSG00000267680, ENSG00000181016, ENSG00000073584, ENSG00000078043, ENSG00000285728, ENSG00000213988, ENSG00000223960, ENSG00000247287, ENSG00000131503, novel.1248, ENSG00000185008, ENSG00000223403, novel.1549, ENSG00000260257, ENSG00000189157, ENSG00000087253, ENSG00000261324, ENSG00000183091, ENSG00000138658, ENSG00000173258, novel.904, ENSG00000215146, ENSG00000281207, ENSG00000247950, ENSG00000107984, ENSG00000280138, ENSG00000220804, ENSG00000213075, novel.380, ENSG00000281183, ENSG00000266173, ENSG00000230551, ENSG00000251432, novel.1597, ENSG00000271976, ENSG00000115414, novel.1591, ENSG00000213468, ENSG00000123552, ENSG00000239653, ENSG00000285106, ENSG00000121210, ENSG00000256433, ENSG00000118407, ENSG00000111799, ENSG00000229692, ENSG00000038427, ENSG00000166263, ENSG00000231999, novel.153, ENSG00000225470, ENSG00000245275, ENSG00000105792, ENSG00000263069, ENSG00000272140, ENSG00000236540, ENSG00000166321, novel.990, ENSG00000265817, ENSG00000228486, ENSG00000273402, ENSG00000259994, ENSG00000224078, ENSG00000074410, ENSG00000224081, ENSG00000198556, novel.583, ENSG00000259158, ENSG00000204778, ENSG00000105664, ENSG00000236778, ENSG00000267370, ENSG00000247828, ENSG00000122420, ENSG00000109576, ENSG00000213742, ENSG00000131711, ENSG00000198625, ENSG00000107890, ENSG00000204514, ENSG00000279722, ENSG00000279520, ENSG00000152078, ENSG00000137965, ENSG00000111339, ENSG00000188234, ENSG00000144810, ENSG00000246859, ENSG00000170236, ENSG00000163660, ENSG00000261556, ENSG00000159708, ENSG00000216895, ENSG00000273018, ENSG00000286699, ENSG00000234290, ENSG00000104237, ENSG00000241343, ENSG00000128833, ENSG00000173200, ENSG00000130224, ENSG00000135205, ENSG00000251259, ENSG00000162601, ENSG00000260853, ENSG00000196757, ENSG00000242193, ENSG00000226149, ENSG00000203872, ENSG00000279059, ENSG00000236194, ENSG00000185946, ENSG00000163728, ENSG00000164197, ENSG00000224138, ENSG00000122481, ENSG00000227934, ENSG00000197961, ENSG00000122477, ENSG00000213949, ENSG00000197168, ENSG00000275481, ENSG00000188321, ENSG00000240038, ENSG00000096093, ENSG00000279518, ENSG00000258441, ENSG00000138468, ENSG00000121570, ENSG00000271009, ENSG00000136010, ENSG00000196460, ENSG00000092969, novel.1005, ENSG00000196381, ENSG00000227543, ENSG00000182993, ENSG00000116001, ENSG00000215158, ENSG00000113356, ENSG00000285752, ENSG00000069431, ENSG00000145348, ENSG00000273271, ENSG00000216937, ENSG00000280067, ENSG00000280099, ENSG00000189007, novel.331, ENSG00000207563, ENSG00000198707, ENSG00000235865, novel.688, ENSG00000152527, ENSG00000234571, novel.420, ENSG00000270953, ENSG00000265298, ENSG00000250596, novel.598, ENSG00000114857, ENSG00000259306, ENSG00000257621, ENSG00000259865, ENSG00000279204, novel.804, ENSG00000248309, ENSG00000236200, ENSG00000269867, ENSG00000272335, ENSG00000248643, ENSG00000260007, ENSG00000259456, ENSG00000181381, ENSG00000243406, ENSG00000167766, ENSG00000110172, ENSG00000244879, ENSG00000082512, ENSG00000197753, ENSG00000110025, ENSG00000261087, novel.533, ENSG00000251562, ENSG00000267481, ENSG00000135164, ENSG00000100897, novel.429, ENSG00000242268, novel.1612, ENSG00000165028, ENSG00000166432, ENSG00000185495, novel.846, ENSG00000244513, ENSG00000244754, ENSG00000259129, ENSG00000267523, ENSG00000258559, ENSG00000154359, ENSG00000229152, ENSG00000233184, ENSG00000232931, ENSG00000008311, ENSG00000280434, novel.340, ENSG00000155657, ENSG00000244055, novel.1551, ENSG00000122417, ENSG00000244733, ENSG00000227671, ENSG00000240280, ENSG00000259248, ENSG00000108846, ENSG00000232656, ENSG00000224660, ENSG00000276855, ENSG00000265917, ENSG00000137878, ENSG00000152580, ENSG00000284862, ENSG00000234810, ENSG00000270091, ENSG00000172955, ENSG00000079134, ENSG00000225205, ENSG00000278949, ENSG00000196295, ENSG00000267002, ENSG00000197580, ENSG00000122674, ENSG00000225670, ENSG00000236526, ENSG00000154258, ENSG00000288569, ENSG00000175265, ENSG00000274525, ENSG00000186105, ENSG00000113600, ENSG00000273373, ENSG00000283445, ENSG00000233967, ENSG00000137819, ENSG00000226252, ENSG00000183281, ENSG00000288596, ENSG00000232995, ENSG00000243716, novel.410, ENSG00000272145, ENSG00000189212, ENSG00000279108, ENSG00000162687, ENSG00000197083, ENSG00000215252, ENSG00000269952, ENSG00000237491, ENSG00000110455, ENSG00000147606, ENSG00000176222, ENSG00000277112, ENSG00000261114, ENSG00000229097, ENSG00000233672, ENSG00000204745, ENSG00000206384, ENSG00000264672, ENSG00000168356, ENSG00000279133, ENSG00000281344, ENSG00000268279, ENSG00000183098, ENSG00000177409, ENSG00000213204, ENSG00000215126, ENSG00000227885, ENSG00000278959, ENSG00000178127, ENSG00000228393, ENSG00000248445, ENSG00000270069, ENSG00000248256, ENSG00000177990, ENSG00000228933, ENSG00000154175, ENSG00000225793, ENSG00000250564, ENSG00000275854, ENSG00000272054, ENSG00000198185, ENSG00000092529, ENSG00000213782, novel.478, ENSG00000272374, novel.1043, ENSG00000132530, ENSG00000280077, ENSG00000281404, ENSG00000046653, ENSG00000258733, ENSG00000175455, ENSG00000283122, ENSG00000170482, ENSG00000280219, ENSG00000286599, ENSG00000230630, novel.231, ENSG00000187753, ENSG00000231551, ENSG00000236039, ENSG00000287778, ENSG00000204177, ENSG00000147138, ENSG00000286555, ENSG00000213139, ENSG00000261799, ENSG00000182389, ENSG00000230590, ENSG00000100483, ENSG00000161912, ENSG00000210194, ENSG00000274422, ENSG00000232934, ENSG00000255389, ENSG00000129521, ENSG00000279456, ENSG00000197670, ENSG00000232295, ENSG00000238018, ENSG00000226029, ENSG00000178386, ENSG00000275367, ENSG00000081052, ENSG00000069493, ENSG00000254860, ENSG00000268205, novel.253, ENSG00000254206, novel.83, ENSG00000229036, ENSG00000258581, novel.978, ENSG00000258168, ENSG00000225920, ENSG00000232593, ENSG00000279636, ENSG00000111837, ENSG00000214827, ENSG00000288585, ENSG00000270039, ENSG00000271870, ENSG00000197332, ENSG00000231607, ENSG00000176563, ENSG00000276334, ENSG00000233237, ENSG00000015479, ENSG00000233029, ENSG00000188738, ENSG00000121101, ENSG00000248254, ENSG00000249721, ENSG00000269124, ENSG00000125551, ENSG00000259668, ENSG00000217684, ENSG00000266171, ENSG00000041982, ENSG00000268189, novel.660, ENSG00000214787, ENSG00000147437, ENSG00000172461, ENSG00000285803, ENSG00000264112, ENSG00000175518, ENSG00000188869, ENSG00000225886, ENSG00000113971, ENSG00000272750, ENSG00000287202, ENSG00000152894, ENSG00000230084, ENSG00000276975, novel.561, ENSG00000233491, ENSG00000254433, ENSG00000271218, ENSG00000196912, ENSG00000163611, ENSG00000250511, ENSG00000281026, ENSG00000260400, novel.245, ENSG00000226239, ENSG00000251273, ENSG00000228363, ENSG00000254054, ENSG00000154898, ENSG00000270055, ENSG00000259556, ENSG00000237513, ENSG00000207741, ENSG00000258521, ENSG00000244161, ENSG00000207721, ENSG00000225507, ENSG00000206530, ENSG00000150637, ENSG00000164176, ENSG00000226803, ENSG00000179909, ENSG00000261625, ENSG00000260423, ENSG00000244676, ENSG00000244687, ENSG00000286207, ENSG00000178440, ENSG00000233379, ENSG00000137948, ENSG00000241975, ENSG00000261098, ENSG00000228060, ENSG00000188739, ENSG00000233327, ENSG00000183171, ENSG00000271533, ENSG00000274615, ENSG00000249502, ENSG00000267313, ENSG00000230650, ENSG00000267758, ENSG00000273055, ENSG00000189423, ENSG00000226690, ENSG00000254343, ENSG00000229618, ENSG00000284747, ENSG00000259030, ENSG00000169203, ENSG00000163806, ENSG00000225313, novel.585, ENSG00000215417, ENSG00000260922, ENSG00000137959, ENSG00000225746, ENSG00000273345, ENSG00000248019, ENSG00000251429, ENSG00000237883, ENSG00000242338, ENSG00000272375, ENSG00000241288, ENSG00000178295, ENSG00000197683, ENSG00000269925, ENSG00000249626, ENSG00000273284, ENSG00000260761, ENSG00000259820, ENSG00000259250, ENSG00000233885, ENSG00000271151, ENSG00000271730, ENSG00000279342, ENSG00000269680, ENSG00000253143, ENSG00000213170, ENSG00000169248, ENSG00000221184, ENSG00000104432, ENSG00000009724, ENSG00000253671, novel.255, ENSG00000248734, ENSG00000272211, novel.643, ENSG00000257176, ENSG00000157404, ENSG00000253667, ENSG00000127364, ENSG00000223519, ENSG00000241322, ENSG00000224468, ENSG00000210174, ENSG00000271843, ENSG00000260118, ENSG00000280776, ENSG00000226312, ENSG00000248174, ENSG00000242028, ENSG00000272037, ENSG00000228492, ENSG00000287134, novel.808, ENSG00000285979, ENSG00000268521, ENSG00000254732, ENSG00000169031, ENSG00000279700, ENSG00000171970, ENSG00000272034, ENSG00000260528, ENSG00000228302, ENSG00000258302, ENSG00000244124, ENSG00000223704, ENSG00000276115, ENSG00000279662, ENSG00000138378, ENSG00000153291, ENSG00000112309, ENSG00000265996, ENSG00000227954, ENSG00000279019, novel.85, ENSG00000244682, ENSG00000265413, novel.1567, novel.865, ENSG00000272010, ENSG00000287248, ENSG00000254165, ENSG00000157578, novel.124, ENSG00000251023, ENSG00000261997, ENSG00000286223, ENSG00000254595, ENSG00000269699, ENSG00000279926, ENSG00000249207, ENSG00000242600, ENSG00000285715, ENSG00000278206, ENSG00000281974, ENSG00000259972, ENSG00000135976, ENSG00000180113, novel.106, ENSG00000278861, novel.1606, ENSG00000187984, ENSG00000197479, ENSG00000286964, ENSG00000119508, ENSG00000266993, ENSG00000272842, ENSG00000224843, ENSG00000188676, ENSG00000279500, ENSG00000252498, ENSG00000153898, ENSG00000274204, ENSG00000230333, ENSG00000123201, novel.1536, ENSG00000166323, ENSG00000232470, ENSG00000272341, ENSG00000279089, ENSG00000259116, ENSG00000248323, novel.647, novel.322, ENSG00000287299, ENSG00000231154, ENSG00000171643, novel.447, ENSG00000118473, ENSG00000286305, ENSG00000251728, ENSG00000285748, ENSG00000234478, novel.249, ENSG00000261136, ENSG00000178852, ENSG00000264176, novel.1527, novel.274, ENSG00000248858, ENSG00000196074, ENSG00000238141, ENSG00000164933, ENSG00000287306, novel.482, ENSG00000248810, novel.1547, ENSG00000267547, novel.1015, ENSG00000250644, ENSG00000260296, ENSG00000279289, ENSG00000228113, ENSG00000226314, ENSG00000233110, ENSG00000165995, ENSG00000269940, ENSG00000261366, ENSG00000151012, ENSG00000261051, ENSG00000281468, ENSG00000238273, ENSG00000137801, ENSG00000274964, novel.1199, ENSG00000256673, ENSG00000278989, ENSG00000279541, ENSG00000273449, ENSG00000272871, ENSG00000260698, ENSG00000253838, ENSG00000239265, ENSG00000112319, ENSG00000273448, ENSG00000132639, ENSG00000286545, ENSG00000247903, ENSG00000153292, ENSG00000112706, ENSG00000271862, ENSG00000172179, ENSG00000286689, ENSG00000268621, ENSG00000225964, ENSG00000270589, novel.562, ENSG00000170160, ENSG00000110427, ENSG00000273243, ENSG00000209082, ENSG00000206869, ENSG00000259562, ENSG00000279432, ENSG00000235888, ENSG00000273891, ENSG00000206658, ENSG00000229127, ENSG00000287944, ENSG00000277767, ENSG00000240244, ENSG00000086288, ENSG00000224431, ENSG00000233750, novel.1518, ENSG00000188626, ENSG00000175967, ENSG00000287986, ENSG00000265791, ENSG00000198838, ENSG00000253939, ENSG00000277687, ENSG00000270347, ENSG00000148053, ENSG00000263990, ENSG00000286652, ENSG00000226476, ENSG00000237975, ENSG00000205359, ENSG00000271553, ENSG00000226245, ENSG00000261187, novel.147, ENSG00000229436, ENSG00000198744, ENSG00000288534, novel.1569, ENSG00000261191, ENSG00000282057, ENSG00000272599, ENSG00000202078, ENSG00000138435, ENSG00000272402, ENSG00000283236, ENSG00000251450, ENSG00000286118, novel.1510, ENSG00000232111, ENSG00000176383, ENSG00000256973, ENSG00000260948, ENSG00000279419, ENSG00000279659, ENSG00000146809, ENSG00000177483, novel.448, ENSG00000235438, ENSG00000287923, ENSG00000278867, ENSG00000267199, ENSG00000091137, novel.645, ENSG00000267595, ENSG00000239704, ENSG00000237672, ENSG00000260618, ENSG00000225213, ENSG00000197816, ENSG00000272370, ENSG00000267152, novel.567, ENSG00000273369, ENSG00000278985, ENSG00000134343, ENSG00000258136, ENSG00000256667, ENSG00000198064, novel.1543, ENSG00000273007, ENSG00000182308, ENSG00000226874, novel.834, ENSG00000245711, ENSG00000229325, ENSG00000232186, ENSG00000254577, ENSG00000229591, ENSG00000234281, ENSG00000244218, ENSG00000249899, ENSG00000223542, ENSG00000264705, ENSG00000166206, ENSG00000270562, ENSG00000252174, ENSG00000165810, ENSG00000279880, ENSG00000260361, ENSG00000227578, ENSG00000260918, ENSG00000246250, ENSG00000264472, ENSG00000278811, ENSG00000270574, ENSG00000236711, ENSG00000288065, ENSG00000250669, ENSG00000225125, ENSG00000269161, ENSG00000270823, ENSG00000116690, ENSG00000273771, ENSG00000269053, ENSG00000237061, ENSG00000280069, ENSG00000266743, ENSG00000230262, ENSG00000146469, novel.1576, ENSG00000229781, ENSG00000274024, ENSG00000279903, ENSG00000285857, ENSG00000275371, ENSG00000225451, ENSG00000211699, ENSG00000223656, ENSG00000227719, ENSG00000288068, ENSG00000255445, novel.526, ENSG00000240216, ENSG00000138109, ENSG00000270031, ENSG00000235308, ENSG00000235427, ENSG00000258636, ENSG00000212452, ENSG00000143318, ENSG00000242516, ENSG00000240522, ENSG00000212384, ENSG00000275580, ENSG00000254694, ENSG00000276957, ENSG00000235724, ENSG00000250337, ENSG00000231851, ENSG00000249835, ENSG00000285804, ENSG00000256499, novel.1517, ENSG00000285704, novel.479, ENSG00000287735, ENSG00000207870, ENSG00000287907, ENSG00000222533, ENSG00000244198, novel.546, ENSG00000229000, ENSG00000227253, ENSG00000230092, ENSG00000273306, ENSG00000172404, novel.629, ENSG00000240322, novel.311, novel.1257, novel.1133, ENSG00000283312, ENSG00000224629, novel.1519, ENSG00000240591, ENSG00000273507, ENSG00000127083, ENSG00000175820, novel.81, novel.126, ENSG00000129167, ENSG00000180139, ENSG00000224216, ENSG00000231845, ENSG00000151655, ENSG00000272128, ENSG00000273650, ENSG00000229816, novel.403, ENSG00000271092, ENSG00000165379, ENSG00000235448, ENSG00000200257, ENSG00000236268, ENSG00000200591, ENSG00000134007, ENSG00000262118, ENSG00000224855, ENSG00000120337, ENSG00000229487, ENSG00000180178, ENSG00000273747, ENSG00000118432, novel.262, ENSG00000188933, ENSG00000271967, ENSG00000221518, novel.1625, ENSG00000230606, ENSG00000099251, ENSG00000206612, ENSG00000285758, ENSG00000166819, ENSG00000288101, novel.630, ENSG00000285159, ENSG00000273338, ENSG00000261684, ENSG00000288067, novel.467, ENSG00000243055, ENSG00000198106, ENSG00000286618, novel.835, ENSG00000273372, ENSG00000255192, ENSG00000252355, ENSG00000225532, ENSG00000259810, ENSG00000246851, ENSG00000275956, ENSG00000285095, ENSG00000272334, ENSG00000277595, ENSG00000207757, ENSG00000237418, ENSG00000286864, ENSG00000285887, ENSG00000264895, ENSG00000153237, ENSG00000219928, ENSG00000228513, ENSG00000246422, ENSG00000199153, ENSG00000278492, ENSG00000269895, ENSG00000199933, ENSG00000269600, ENSG00000253790, ENSG00000287188, ENSG00000254397, ENSG00000225101, ENSG00000207825, ENSG00000266126, ENSG00000278456, ENSG00000275413, ENSG00000206888, ENSG00000207291, ENSG00000239997, ENSG00000226186, ENSG00000240666, ENSG00000201444, ENSG00000200648, ENSG00000273012, ENSG00000252711, ENSG00000170558, ENSG00000218792, ENSG00000286378, ENSG00000285692, ENSG00000232265, ENSG00000253197, ENSG00000145777, ENSG00000205822, ENSG00000201499, ENSG00000235397, ENSG00000258876, novel.1421, novel.1568, ENSG00000182050, ENSG00000252620, ENSG00000170074, novel.1342, ENSG00000189014, ENSG00000213695, novel.164, ENSG00000279301, ENSG00000270147, novel.604, ENSG00000229236, ENSG00000248307, ENSG00000277948, ENSG00000268223, ENSG00000212443, ENSG00000267707, |
